# Implementation of meiosis prophase I programme requires a conserved retinoid-independent stabilizer of meiotic transcripts

**DOI:** 10.1038/ncomms10324

**Published:** 2016-01-08

**Authors:** Emilie Abby, Sophie Tourpin, Jonathan Ribeiro, Katrin Daniel, Sébastien Messiaen, Delphine Moison, Justine Guerquin, Jean-Charles Gaillard, Jean Armengaud, Francina Langa, Attila Toth, Emmanuelle Martini, Gabriel Livera

**Affiliations:** 1 Université Paris Diderot, Sorbonne Paris Cité, Laboratory of Development of the Gonads, Unit of Stem Cells and Radiation, UMR-967, BP 6, Fontenay-aux-Roses 92265, France; 2 CEA, DSV, iRCM, SCSR, LDG, Fontenay-aux-Roses 92265, France; 3 INSERM, Unité 967, Fontenay-aux-Roses F-92265, France; 4 Université Paris-Sud, UMR-967, Fontenay-aux-Roses F-92265, France; 5 Molecular Cell Biology Group/Experimental Center, Institute of Physiological Chemistry, Medical School, MTZ, Dresden University of Technology, Fiedlerstrasse 42, Dresden 01307, Germany; 6 CEA, DSV/IBITEC-S/SPI/Li2D, Laboratory ‘Innovative Technologies for Detection and Diagnostic', CEA-Marcoule, BP 17171, Bagnols-sur-Cèze F-30200, France; 7 Centre d'Ingénierie Génétique Murine, Institut Pasteur, Paris 75015, France

## Abstract

Sexual reproduction is crucially dependent on meiosis, a conserved, specialized cell division programme that is essential for the production of haploid gametes. Here we demonstrate that fertility and the implementation of the meiotic programme require a previously uncharacterized meiosis-specific protein, MEIOC. *Meioc* invalidation in mice induces early and pleiotropic meiotic defects in males and females. MEIOC prevents meiotic transcript degradation and interacts with an RNA helicase that binds numerous meiotic mRNAs. Our results indicate that proper engagement into meiosis necessitates the specific stabilization of meiotic transcripts, a previously little-appreciated feature in mammals. Remarkably, the upregulation of MEIOC at the onset of meiosis does not require retinoic acid and STRA8 signalling. Thus, we propose that the complete induction of the meiotic programme requires both retinoic acid-dependent and -independent mechanisms. The latter process involving post-transcriptional regulation likely represents an ancestral mechanism, given that MEIOC homologues are conserved throughout multicellular animals.

## Abstract (web summary)

Meiosis is a cell division program that produces haploid gametes and is initiated by a retinoic acid-dependent process. Here the authors report that a meiosis-specific protein, MEIOC, is upregulated in a retinoic acid-independent manner and is required to stabilise meiosis-specific transcripts.

## Graphical abstract

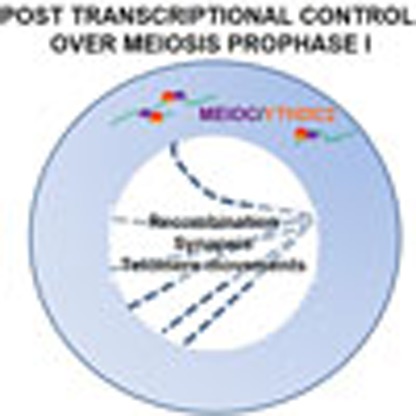

Meiosis is a core event of sexual reproduction. The premeiotic DNA replication followed by meiosis prophase I (MPI) are the first critical stages of the meiotic process. During these stages, the meiotic programme diverges from the mitotic programme, and multiple meiosis-specific features are coordinately implemented to prepare for the later orderly halving of the genome^1^. Thus, the production of healthy haploid gametes requires tight control of the meiotic initiation in the germline.

Entry into meiosis can be defined both through a drastic change in gene expression and through initiation of the nuclear events of the MPI. MPI is relatively long and is typically divided into four successive stages: leptotene, zygotene, pachytene and diplotene, which are preceded by the preleptotene stage, a specialized S phase. Recent advances have highlighted an extraordinary complexity of MPI that requires compacting chromatin, pairing homologous chromosomes, splitting the DNA, specialized recombination and telomere movements^2^,^3^,^4^. Thus, it is no surprise that specialized machinery is needed corresponding to numerous meiosis-specific proteins, with the expression of the corresponding genes being specifically upregulated at the onset of MPI.

In mammals, all female germ cells initiate meiosis during fetal life, whereas male germ cells enter meiosis regularly throughout postnatal life^5^. In the mouse embryonic ovary, all germ cells switch abruptly from mitosis to meiosis between 13.5 and 15.5 days post conception (d.p.c.). In the male mouse, however, gonocytes resume proliferation just after birth, differentiate into proliferating spermatogonia and then initiate meiosis at ∼8 days post-partum (d.p.p.)^6^. The gonadal somatic environment governs this sexual dichotomy. A widely held view proposes that retinoic acid (RA) is the external signal that triggers meiotic entry through the upregulation of the *Stimulated by retinoic acid 8* gene (*Stra8*). *Stra8* is currently the sole known gatekeeper of the mitotic/meiotic switch in female and male vertebrates^7^,^8^. It is expressed at the preleptotene stage and is required for the proper induction of many, but not all, meiosis-specific genes^9^,^10^,^11^,^12^. Although the exact function of STRA8 is currently unknown, it has been proposed to have transcriptional activation potential^13^,^14^. Conflicting data exist regarding its absolute requirement for the proper initiation of cellular events of the MPI. Indeed, genetic models have proposed that *Stra8*^*−/−*^ germ cells either do not preform premeiotic DNA replication and the subsequent steps^11^,^15^ or that they do so and initiate early prophase I to arrest shortly after^10^.

As a whole, the regulation of the meiotic programme in mammals remains a matter of debates. Although post-transcriptional regulation is well known to play important roles in the execution of late meiotic and sex-specific post-meiotic processes, its role in meiosis entry and progression through MPI has not been appreciated in mammals^16^,^17^. Here we identify ‘Meiosis specific with Coiled-coil domain' (MEIOC) as a critical factor for both the correct execution of early meiotic events and the stability of early meiotic RNA messengers. Thus, we propose that post-transcriptional control of mRNA stability is key to the implementation of the meiotic programme.

## Results

### MEIOC is conserved through evolution

**Figure 1:**
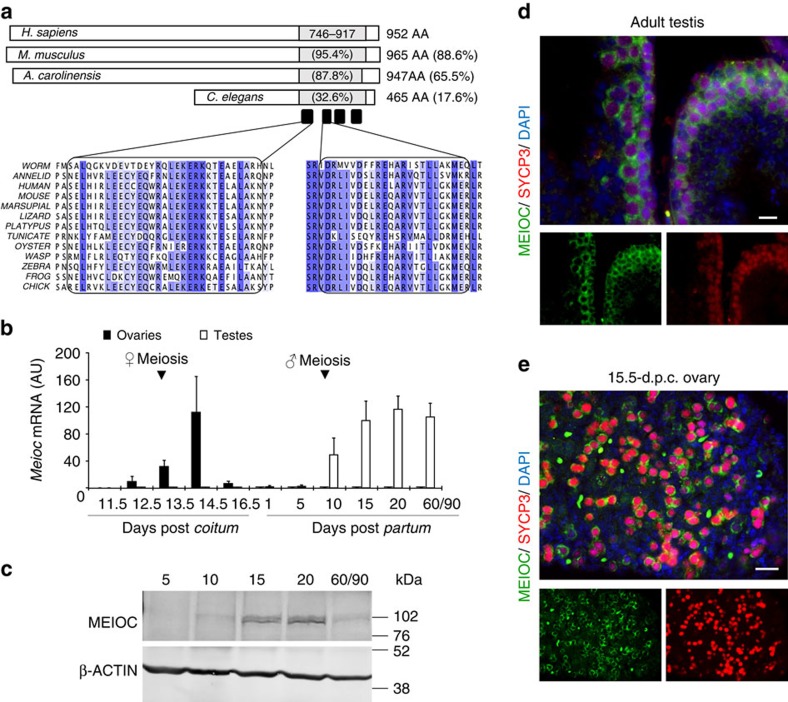
*Meioc* is a conserved and meiosis prophase I-specific gene. (**a**) Schematic representation of MEIOC proteins in the indicated species (see also Supplementary Fig. 1). A single conserved domain of unknown function (DUF4582, grey boxes) is retrieved in the sequence of all proteins. It features a highly conserved coiled-coil domain composed of four helixes (black boxes). The length and percentage of identity of the complete sequence compared with that of humans are indicated on the right, and the identities of the coiled-coil domain are indicated in the grey boxes. Comparison of amino-acid sequences of helixes 1 and 2 are detailed for the indicated species. Blue indicates conservation (Clustal2.1). Represented species are as follows: worm, *Caenorhabditis elegans*; tunicate, *Ciona intestinalis*; wasp, *Nasonia vitripennis*; annelid, *Capitella teleta*; oyster, *Crassostrea gigas*; urchin, *Strongylocentrotus purpuratus*; zebrafish, *Danio rerio*; frog, *Xenopus tropicalis*; platypus, *Ornithorhynchus anatinus*; lizard, *Anolis carolinensis*; marsupial, *Sarcophilus harrisii*; mouse, *Mus musculus*; rat, *Rattus norvegicus*; human, *Homo sapiens*; and chick, *Gallus gallus*. (**b**) *Meioc* mRNA expression measured by RT–qPCR in whole fetal and postnatal mouse ovaries (black columns) and testes (white columns) at the indicated developmental stages. Arrows indicate meiosis initiation. Mean±s.e.m., *n*=3 pools of 3–10 gonads. (**c**) Western blot analysis of MEIOC in mouse testis protein extracts at the indicated postnatal ages (days post-partum). β-actin/ACTB was used as a control. (**d**,**e**) Immunofluorescence for MEIOC (green), SYCP3 (red) and DAPI (blue) in (**d**) adult wild-type testis sections (scale bar, 20 μm) and (**e**) fetal (15.5 d.p.c.) wild-type ovaries (scale bar, 20 μm).

To identify new candidate meiotic genes, we exploited the developmental dichotomy in embryonic germ cells when female ones enter meiosis but male ones do not. By analysing several sets of transcriptomic data^18^,^19^,^20^, we identified one candidate gene among those with the highest sexually differential expression during embryonic gonad development. This nucleotide sequence (previously identified as *Gm1564*) corresponded to eight putative exons located on chromosome 11 of the mouse genome and potentially encodes a protein of 965 amino acids of hitherto unknown function. Homologues were identified in the genomes of all vertebrates and of most invertebrates, with the notable exception of drosophila and unicellular organisms, demonstrating that this gene is highly evolutionarily conserved (Fig. 1a, Supplementary Fig. 1 and Supplementary Table 1). A comparison of these putative proteins led to the identification of a coiled-coil domain with a very high degree of conservation (95% mouse and human) in the C-terminal region of the putative protein (Fig. 1a). We thus decided to term this gene *Meioc* for *Meiosis specific with coiled-coil domain*.

### *MEIOC* is expressed in germ cells during meiotic prophase I

A detailed expression profile of *Meioc* mRNA in mouse tissues revealed that *Meioc* was exclusively detected in the fetal ovary and postnatal and adult testes (Fig. 1b), but not in somatic tissues (Supplementary Fig. 2a,b). In the ovary, *Meioc* appeared at ∼13.5 d.p.c. and disappeared shortly thereafter. In the postnatal testis, *Meioc* expression rose between 5 and 10 d.p.p. and was maintained throughout adulthood. Germ cell sorting using fetal ovaries from OCT4-GFP embryos demonstrated that *Meioc* was germ cell-specific (Supplementary Fig. 2c). These expression profiles correlated with the timing of early meiotic events. This correlation was also observed in the human fetal ovary in which *MEIOC* expression started at 11 weeks post fertilization (Supplementary Fig. 2d) when meiosis was initiated based on *STRA8* detection.

**Figure 2:**
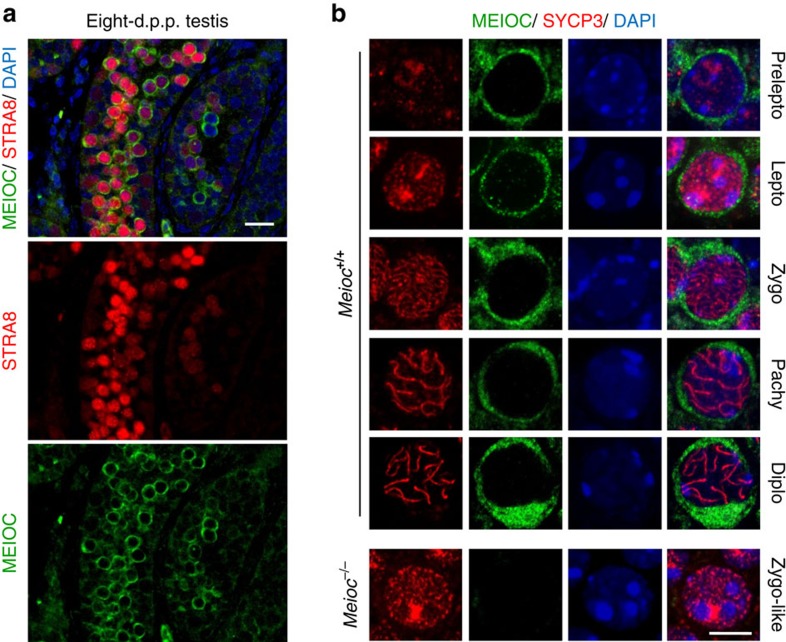
MEIOC protein is expressed in germ cells from the onset of meiosis. (**a**) Immunofluorescence staining for MEIOC (green), STRA8 (red) and DAPI (blue) in 8 days post-partum (d.p.p.) testis sections. Scale bar, 20 μm. (**b**) Confocal acquisitions of representative *Meioc*^*+/+*^ and *Meioc*^*−/−*^ spermatocytes stained for SYCP3 (red), MEIOC (green) and DAPI (blue). Diplo, diplotene; Lepto, leptotene; Pachy, pachytene; Prelepto, preleptotene; Zygo, zygotene. Scale bar, 5 μm.

The corresponding protein was detected using western blot analysis in the mouse postnatal testis, and immunofluorescence staining localized the protein to the cytoplasmic compartment of female and male meiocytes (Fig. 1c–e). The protein was detected at the expected molecular weight (106 kDa) with various sera and antibodies validated against a recombinant protein and *Meioc*^*−/−*^ testis (Supplementary Fig. 3 and see later). To gain better insight into the role of MEIOC we detailed its expression during spermatogenesis. Premeiotic stage and the first stage of MPI, termed the leptotene, are characterized by the presence of STRA8 protein^9^. In the postnatal testis, most (88%) of the STRA8-positive cells also expressed MEIOC, indicating that MEIOC was present at the onset of meiosis (Fig. 2a). This observation was performed at 8 d.p.p. when the first meiotic wave started and at older ages (for example, 10 d.p.p. and adult, Fig. 10c,d). As spermatocytes enter and progress throughout MPI stages (Fig. 2b), which are recognized based on the appearance of the SYCP3 protein^21^, MEIOC staining was observed from preleptotene to diplotene stage. Similar pattern was observed in female germ cells at MPI (Supplementary Fig. 3c). We thus concluded that MEIOC is a new factor specific to the early steps of meiosis.

### Meioc is required for fertility and meiosis completion

**Figure 3:**
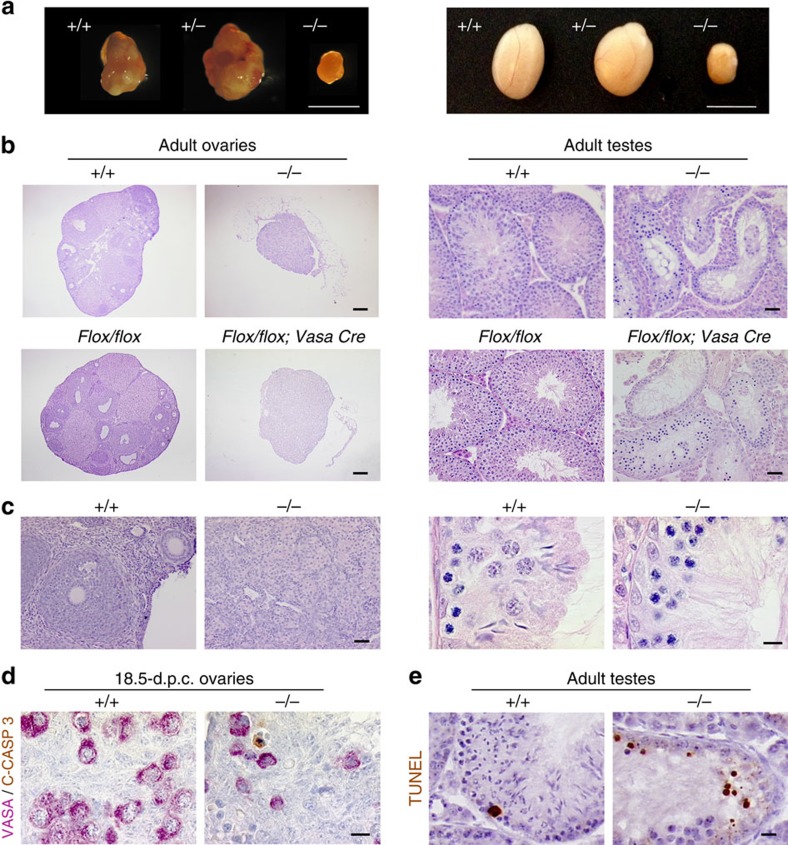
*Meioc* is required for fertility and meiosis completion. (**a**) Testes and ovaries from adult *Meioc*^*−/−*^ mice (knockout straight) are significantly smaller in size than wild type and heterozygous gonads. Scale bars, 2 mm (left) and 5 mm (right). (**b**) Left: histological sections of adult *Meioc*^*+/+*^, *Meioc*^*−/−*^(knockout straight), *Meioc*^*flox/flox*^ and *Meioc*^*flox/flox*^*;Vasa Cre* ovaries. Scale bar, 200 μm. Right: histological sections of *Meioc*^*+/+*^, *Meioc*^*−/−*^*, Meioc*^*flox/flox*^ and *Meioc*^*flox/flox*^*;Vasa Cre* adult testes. Scale bar, 40 μm. (**c**) Representative magnifications of histological sections of adult *Meioc*^*+/+*^, *Meioc*^*−/−*^ (left) ovaries and (right) testes. Scale bar, 40 and 10 μm. (**d**) Histological sections of 18.5-d.p.c. *Meioc*^*+/+*^ and *Meioc*^*−/−*^ ovaries stained for cleaved-caspase 3 (C-CASP 3) and VASA/DDX4. Scale bar, 10 μm. (**e**) Histological sections of adult *Meioc*^*+/+*^ and *Meioc*^*−/−*^ testes stained for TUNEL. Scale bar, 20 μm.

**Figure 4:**
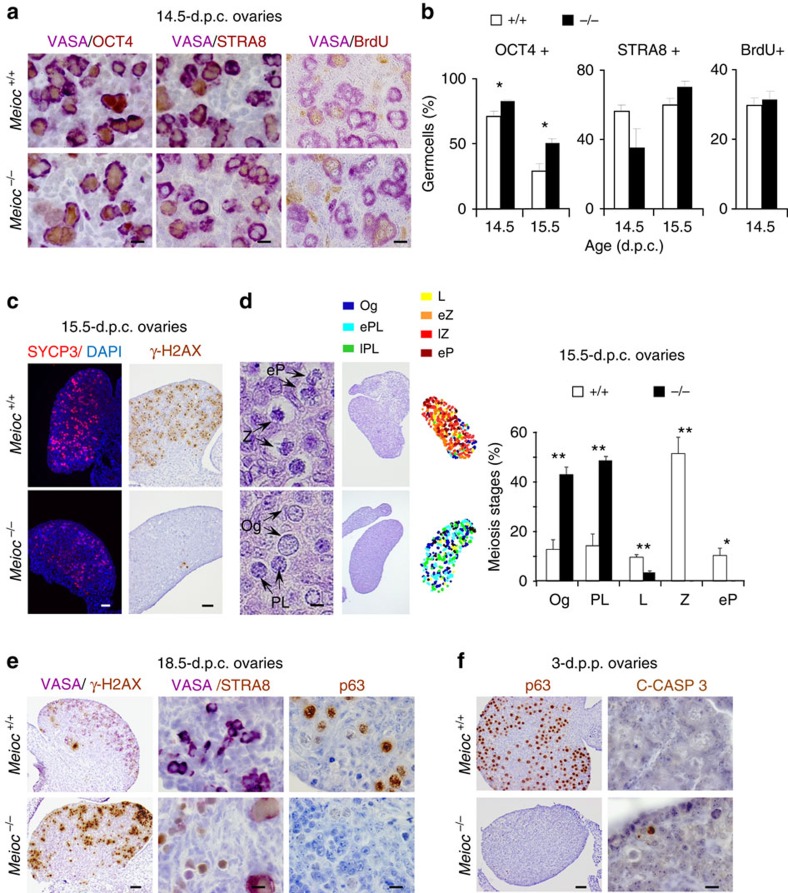
Early meiotic defects in *Meioc*^*−/−*^ ovaries. (**a**) Histological sections of 14.5-d.p.c. *Meioc*^*+/+*^ and *Meioc*^*−/−*^ ovaries stained for VASA/DDX4 and OCT4/POU5F1, STRA8 or BrdU. Scale bar, 10 μm. OCT4 identified oogonia, and STRA8 labelled pre-meiotic cells. BrdU was injected to pregnant females 3 h before harvesting fetal gonads. BrdU incorporation revealed replicating cells. (**b**) The percentage of cells positive for the indicated marker of interest and for VASA, a germ cell marker, was determined in 14.5- and 15.5-d.p.c. *Meioc*^*+/+*^ (white columns) and *Meioc*^*−/−*^ (black columns) ovaries. Mean±s.e.m. At least 200 cells were counted per gonad analysed. *n*=3 embryos analysed; *P<0.05 (Student's *t*-test). (**c**) Histological sections of 15.5-d.p.c. *Meioc*^*+/+*^ and *Meioc*^*−/−*^ ovaries stained for SYCP3 and γ-H2AX meiotic markers showing delayed meiosis entry and progression in *Meioc*-deficient ovaries. Scale bar, 40 μm. (**d**) Meiosis prophase I stages in *Meioc*^*+/+*^ and Meioc^*−/−*^ 15.5-d.p.c. ovaries. e, early; l, late; L, leptotene; Og, oogonia; P, pachytene; PL, preleptotene; Sg, spermatogonia; Z, zygotene. Mean±s.e.m., *n*=3 mice analysed; *P<0.05; **P<0.01 (Student's *t*-test). Scale bar, 5 μm. (**e**) Histological sections of 18.5-d.p.c. *Meioc*^*+/+*^ and *Meioc*^*−/−*^ ovaries stained for γ-H2AX and VASA, STRA8 and VASA or P63. Scale bar, 40 and 10 μm. (**f**) Histological sections of 3-d.p.p. *Meioc*^*+/+*^ and *Meioc*^*−/−*^ovaries stained for P63 or C-CASP 3. Scale bar, 40 and 10 μm.

To gain further insight into the biological function of MEIOC, we generated a corresponding null mutant mouse (Supplementary Fig. 4a). This was performed by taking advantage of the Knockout Mice Project (KOMP) repository of targeted embryonic stem cells because of the insertion of a promoter-less lacZ reporter between exons 2 and 3 of the *Meioc* gene. This produced a knockout (KO) straight allele because of interruption of the *Meioc* gene. Homozygous mutant mice were obtained at the expected Mendelian ratio and were viable. In those animals, MEIOC mRNA and protein appeared to be undetectable, confirming that we indeed generated a null mutant (Supplementary Fig. 4b,c). Homozygous mutant mice grew normally but were fully infertile and contained barely detectable ovaries and small testes at adulthood (that is, mutant testes were 75% smaller than wild-type testes, Fig. 3 and Supplementary Fig. 4f). A histological examination indicated a complete absence of follicles in the ovaries, as well as an absence of spermatozoon in the seminiferous tubules and epididymides (Fig. 3b,c and Supplementary Fig. 4d,e). Removing the insertion cassette interrupting the *Meioc* gene with a flippase created a floxed exon 3 that could be further excised with cre recombinase. The morphology of the gonads of *Meioc*^*Flox/Flox*^ animals was similar to that of the wild types, indicating that the *Meioc* mutation was the solely responsible for the fertility defect. Deletion of *Meioc* exon 3 created with *Vasa/Ddx4*-cre generated a deleted allele (Δ) specifically in fetal germ cells and induced exactly the same gonadal defects as KO straight *Meioc* mice (Fig. 3b and Supplementary Fig. 4a). This result demonstrated that infertility depended exclusively on the role of *Meioc* in germ cells. The KO straight mice, later referred to as *Meioc*^*−/−*^, were used for the rest of the study. Histological analysis of the postnatal and adult *Meioc*^*−/−*^ ovaries revealed that this tissue was totally devoid of oocytes with a complete germ cell loss at adulthood (Fig. 3c) that was already observed at 8 d.p.p. (Supplementary Fig. 4h). Accordingly, during the perinatal period, germ cell density was greatly reduced with numerous *Meioc*^*−/−*^ oocytes stained with apoptotic markers (Figs 3d and 4f). In the adult mutant testis, ∼60% of the tubules contained spermatocytes arrested early during MPI (that is, preleptotene, leptotene and zygotene cells) and the remaining tubules contained only spermatogonia (Fig. 3b,c and Supplementary Fig. 4e). In addition, numerous apoptotic cells labelled with terminal deoxynucleotidyl transferase dUTP nick end labelling (TUNEL) assay were detected in the tubules of the *Meioc*^*−/−*^ mice (Fig. 3e and Supplementary Fig. 4g). Altogether, these results indicated that *Meioc* is mandatory for MPI completion in both males and females.

### Early meiotic defects in Meioc^−/−^ mice

To further investigate the reason for the absence of oocytes in the postnatal ovary, we performed a meticulous analysis during female gonad development. OCT4 downregulation, STRA8 upregulation and 5-bromo-2′-deoxyuridine (BrdU) incorporation reflect the premeiotic events in female germ cells^5^,^22^. These appeared only mildly affected in *Meioc*^*−/−*^ 14.5 d.p.c. ovaries (Fig. 4a,b). In wild-type fetal ovaries, most germ cells entered meiosis at 15.5 d.p.c., as indicated by the detection of both SYCP3 and the marker for DNA double-strand breaks (DSB), γ-H2AX (ref. 23; Fig. 4c). These meiotic markers were poorly detected at this developmental stage in the *Meioc*^*−/−*^ ovaries, although germ cell density appeared unaltered (Fig. 4c). A precise quantification of germ cells present in the 15.5 d.p.c. ovary based on chromatin features revealed numerous preleptotene cells in *Meioc*^*−/−*^, whereas in the wild type most germ cells rapidly progressed to the zygotene stage (Fig. 4d). Later during fetal life in the wild-type ovaries, STRA8 disappeared shortly after meiotic entry, the γ-H2AX signal was progressively lost and P63 was upregulated, indicating the completion of the pachytene stage^24^,^25^ (Fig. 4e). In 18.5-d.p.c. *Meioc*^*−/−*^ ovaries, numerous germ cells remained positive for STRA8, an abundance of γ-H2AX was detected, a SYCP3 staining typical of early MPI was observed and P63 was undetectable (Fig. 4e and Supplementary Fig. 3c). This was confirmed with the observation of cells resembling preleptotene, leptotene and zygotene stages in haematoxylin and eosin-stained sections (Supplementary Fig. 4i). This indicated that, ultimately, some germ cells entered meiosis later during fetal life, but these poorly progressed along MPI and none reached the end of prophase I. In 3-d.p.p. *Meioc*^*−/−*^ ovaries, no oocytes were detected based on the P63 marker and numerous germ cells were stained with the marker for apoptosis, cleaved-caspase 3 (Fig. 4f). Thus, in the absence of *Meioc*, meiosis progression is strongly delayed in female germ cells starting from the preleptotene stage with an early arrest during MPI leading to oocytes loss.

**Figure 5:**
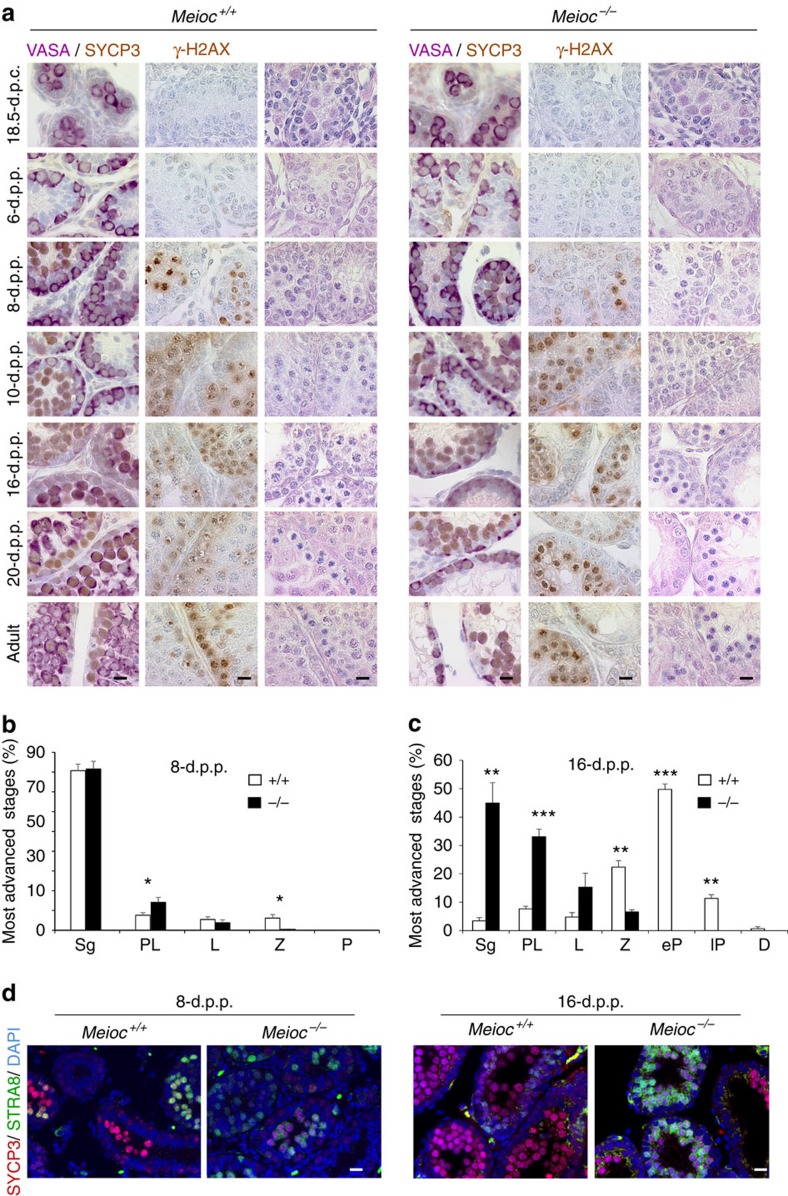
Early meiotic defects in *Meioc*^*−/−*^ testes. (**a**) Histological sections of *Meioc*^*+/+*^ and *Meioc*^*−/−*^ testes at the indicated ages stained for VASA and SYCP3 or γ-H2AX or haematoxylin and eosin. d.p.c., days post conception; d.p.p., days post-partum. Scale bar, 10 μm. (**b**,**c**) Spermatogonial and meiosis prophase I stages in *Meioc*^+/+^ (white columns) and ^*−/−*^ (black columns). (**b**) Eight-d.p.p. and (**c**) sixteen-d.p.p. testes. D, Diplotene; e, early; l, late; L, leptotene; P, pachytene; PL, preleptotene; Sg, spermatogonia; Z, zygotene. Mean±s.e.m., mice analysed *n*=3–7, *P<0.05; **P<0.01; ***P<0.001 (Student's *t*-test). (**d**) Immunofluorescence staining for SYCP3 (red), STRA8 (green) and DAPI (blue) in *Meioc*^*+/+*^ and *Meioc*^*−/−*^ (left) 8-d.p.p. and (right) 16-d.p.p. testis sections. Scale bars, 20 μm.

**Figure 6:**
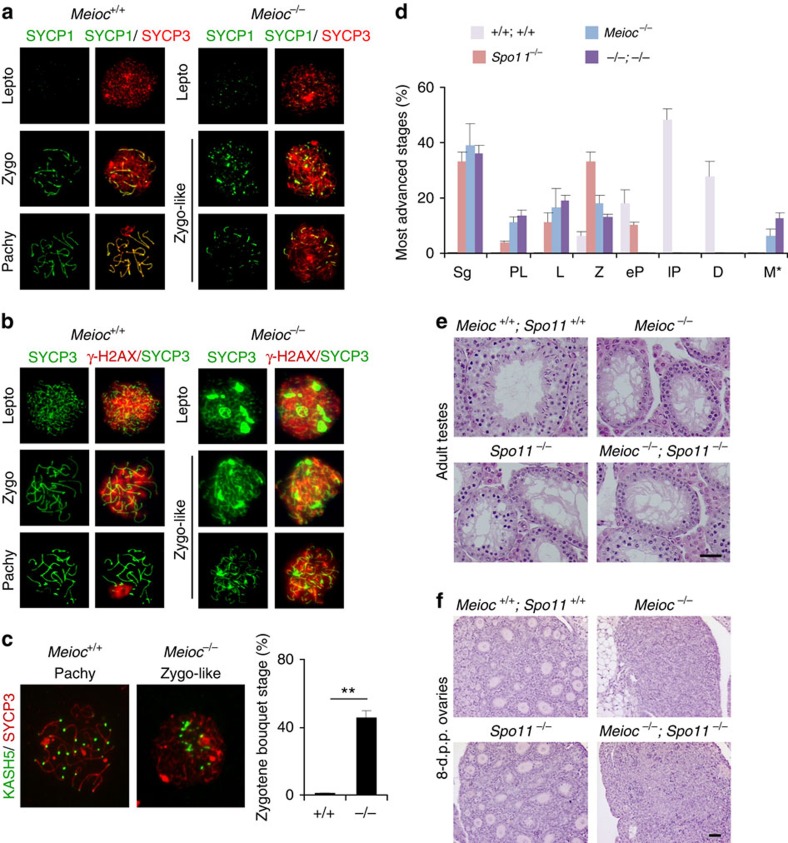
Pleiotropic meiotic defects in *Meioc-*deficient spermatocytes. (**a**,**b**) *Meioc*^+/+^ and *Meioc*^*−/−*^ spermatocyte chromosome spreads at the indicated meiosis prophase I stages stained for (**a**) SYCP1 (green) and SYCP3 (red) or (**b**) γH2AX (red) and SYCP3 (green). Lepto, leptotene; Pachy, pachytene; Zygo, zygotene. (**c**) Representative *Meioc*^+/+^ and *Meioc*^*−/−*^ spermatocyte chromosome spreads at pachytene- and zygotene-like stages stained with SYCP3 (red) and KASH5 (green; meiotic telomere marker). A graph shows the proportion of cells with clustered telomeres (bouquet) among zygotene cells in adult testes. This suggests an extensive duration of the bouquet stage, a transitory telomere clustering occurring during zygotene stage. Mean±s.e.m., mice analysed *n*=3, **P<0.01 (Student's *t*-test). (**d**) Quantification of meiosis prophase I most advanced stages per tubule in adult testes revealed that *Spo11*^*−/−*^ spermatocytes progress until the pachytene-like stage, whereas *Meioc* invalidation induces arrest at a zygotene-like stage and abnormal metaphases, regardless of the genotype. D, Diplotene; e, early; l, late; L, leptotene; M*, abnormal metaphases; P, pachytene; PL, preleptotene; Spg, spermatogonia; Z, zygotene. Mean±s.e.m.; *n*=3 mice analysed per genotype. For each cell type analysed, statistical comparison (analysis of variance, ANOVA) indicated no significant differences between *Meioc*^*−/−*^ and *Meioc*^*−/−*^;*Spo11*^*−/−*^. (**e**) Histological sections of adult testis from wild type (*Meioc*^*+/+*^*; Spo11*^+/+^), *Spo11*^*−/−*^, *Meioc*^*−/−*^ and *Meioc*^*−/−*^;*Spo11*^*−/−*^ mice. Scale bar, 40 μm. (**f**) Histological sections of 8-d.p.p. ovaries from wild type, *Meioc*^*−/−*^, *Spo11*^*−/−*^ and *Meioc*^*−/−*^*; Spo11*^*−/−*^ females. Scale bar, 40 μm.

We next performed similar studies in the postnatal testis to define the kinetics of appearance of the male meiotic defects as a function of age (Fig. 5). As expected, in wild-type testis, at 6 d.p.p. only spermatogonia were present. At 8 d.p.p., the first spermatogenic wave initiated and meiotic cells appeared in a fraction of the tubules based on SYCP3 and γ-H2AX stainings. At 10 and 16 d.p.p., MPI progressed, respectively, to zygotene and pachytene stages and was progressively initiated in all seminiferous tubes in wild-type testes^26^,^27^. At 20 d.p.p., we observed the first spermatocytes that completed MPI with the presence of meiotic metaphases and round spermatids. *Meioc*^*−/−*^ testis presented no developmental defects at 18.5 and 6 d.p.p. with a normal abundance of mitotic germ cells (Fig. 5a). At 8 d.p.p., when meiosis initiates, meiotic markers γ-H2AX and SYCP3 appeared to be globally weaker in *Meioc*^*−/−*^ than in *Meioc*^*+/+*^ testes (Fig. 5a). At later ages, we observed a progressive accumulation of γ-H2AX- and SYCP3-positive cells. Quantification of spermatocytes on the basis of chromatin shape and compaction revealed an accumulation of preleptotene cells and less abundant zygotene cells when compared with *Meioc*^*+/+*^ testes (Fig. 5b,c). At 8 d.p.p., the increased number of preleptotene cells fits with the weak stainings for meiotic markers observed. This was further confirmed with SYCP3/STRA8 co-immunostaining indicating a relatively normal abundance of STRA8 but weaker SYCP3 staining and notably fewer cells stained for only SYCP3, reflecting late leptotene and zygotene cells (Fig. 5d). At 16 d.p.p., we observed a clear accumulation of preleptotene cells and this was confirmed by an abundance of cells that still expressed STRA8 (Fig. 5c,d). BrdU incorporation assays performed at this age indicated no overt defect of pre-meiotic DNA replication (Supplementary Fig. 7c). Analysis of adult spermatocytes in *Meioc*^*−/−*^ testes revealed that those that succeeded in engaging meiosis arrested mostly at leptotene or early zygotene-like stages (Fig. 6d). Thus, the male meiotic defect in *Meioc* mutants appeared with the first meiotic cells and was similar throughout the various spermatogenic waves. Regardless of sex or genetic background, we never observed any pachytene cells in *Meioc* mutant mice. Overall, early meiotic events including initiation and progression were affected in *Meioc*^*−/−*^ testes.

### Pleiotropic meiotic defects in Meioc^*−/−*^ mice

To address the underlying reason for the early meiosis arrest in males, we investigated the MPI-linked process in *Meioc*-deficient mice in depth using chromosome spreads from 16-d.p.p. and adult testes (Fig. 6). The meiotic arrest was especially apparent through analysis of the synaptonemal complex (SC) morphological development. Formation of the SC along homologous chromosomes during meiosis is required for the proper completion of synapsis and recombination events^28^,^29^. Most *Meioc*^*−/−*^ spermatocytes were similar to preleptotene and leptotene stages with partial and discontinuous SYCP3 staining along the chromosome axes; occasionally, cells progressed further to display continuous SYCP3 along chromosome axes and small stretches of SYCP1, a late component of the central element^29^ (Fig. 6a). Specialized cohesion complexes are essential during meiosis for proper assembly of the SC^30^. The loading of the cohesins SMC3 and REC8 appeared unaffected by *Meioc* invalidation (Supplementary Fig. 5a,b). During MPI, numerous DSBs are created and repaired to allow the pairing of homologous chromosomes and meiotic recombination^2^,^3^. In *Meioc*^*−/−*^ testis, all spermatocytes were positive for γ-H2AX, which could indicate both a DSB repair defect and a synapsis defect (Fig. 6b and Supplementary Fig. 4e). To specifically monitor the recombination process, we counted RAD51 and DMC1 recombinases and RPA2 foci that marked early recombination intermediates or unrepaired DSBs (Supplementary Fig. 6a–c). We found that these foci formed normally but persisted in *Meioc*^*−/−*^ meiocytes indicating defective DSB repair. To determine whether *Meioc*^*−/−*^ defects were directly linked to DSB formation and repair, we characterized the effect of *Meioc* invalidation in absence of programmed DSB in a *Spo11*^*−/−*^ background^31^. Both male and female *Meioc*^*−/−*^*;Spo11*^*−/−*^ double mutants were phenotypically similar to the *Meioc* mutants (Fig. 6d–f). This indicated that the meiotic defects in *Meioc*^*−/−*^ animals do not rely exclusively on DSB repair and reflect a wider deregulation of the meiotic programme.

A key feature of meiotic recombination and MPI is the attachment of telomeres to the KASH5 (KASH domain protein 5 also known as coiled-coil domain containing 155) protein containing *trans*-nuclear envelope protein complexes^32^,^33^. This results in the transient clustering of telomeres during the leptotene and early zygotene and allows the active movement of telomeres by cytoplasmic motors, which is critical for efficient pairing of homologous chromosomes and progression through prophase^4^,^34^. KASH5 staining was observed at telomeric sites in *Meioc*-deficient spermatocytes suggesting normal attachment of telomeres to the nuclear envelope. However, in approximately half of the zygotene-like cells, KASH5 foci remained clustered indicating an arrest at the so-called ‘bouquet' stage (Fig. 6c). Similar data indicating a persistent clustering of the telomeres in *Meioc*^*−/−*^ spermatocytes were obtained with CREST (centromere being acrocentric in the mouse; Supplementary Fig. 5c). However, one cannot conclude that there is a specific problem with telomere movement, as such clustering might merely reflect defects in several pathways (that is, the lack of DSB repair)^35^. Altogether, *Meioc*-deficient spermatocytes that initiated meiosis displayed multiple deficiencies including defects in SC formation and DSB repair, likely explaining the arrest in MPI.

### Abnormal metaphases in Meioc^*−/−*^ gonads

**Figure 7:**
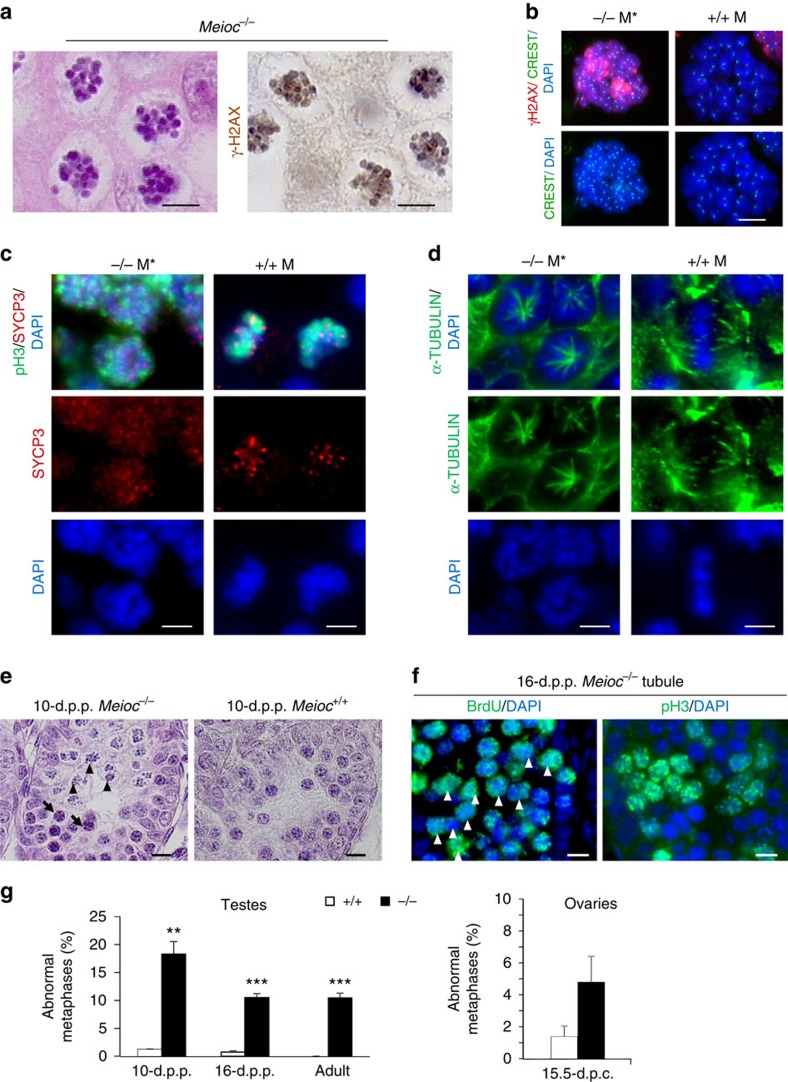
Abnormal metaphase phenotype in *Meioc-*deficient gonads. (**a**) Magnification of abnormal metaphases in 16-d.p.p. *Meioc*^*−/−*^ testis histological sections stained for haematoxylin and eosin (left) or γ-H2AX (right). Scale bar, 5 μm. (**b**) Magnification of chromosome spreads of meiotic metaphase I in Meioc^+/+^ and abnormal metaphase in *Meioc*^*−/−*^ testes stained with DAPI, CREST and γ-H2AX. Scale bar, 5 μm. (**c**,**d**) Sixteen-d.p.p. Meioc^*−/−*^ and adult *Meioc*^*+/+*^ testis sections were stained for (**c**) pH3 (green)/SYCP3 (red)/DAPI (blue) or for (**d**) α-TUBULIN (green) and DAPI (blue). Scale bar, 5 μm. M, metaphase; M*, abnormal metaphase. Similarly to meiotic metaphases, abnormal metaphases in *Meioc* mutants display SYCP3 staining but they are distinguished because of atypical rosette shape and hemispindle. (**e**) Representative histological sections of 10-d.p.p. *Meioc*^*−/−*^ and *Meioc*^*+/+*^ testes showing preleptotene cells associated with abnormal metaphases in the mutant testes. Arrowheads indicate abnormal metaphases; arrows indicate preleptotene cells. Scale bar, 10 μm. (**f**) Magnification of abnormal metaphases and associated cells in a *Meioc*^*−/−*^ 16-d.p.p. seminiferious tubule. Adjacent sections were stained for BrdU or pH3 (green) and DAPI (blue). BrdU was injected to 15-d.p.p. *Meioc*^*−/−*^ mouse 36 h before harvesting the gonads. The presence of BrdU in the abnormal metaphases suggests that these cells were at the preleptotene stage before reaching a metaphase-like stage. Arrowheads indicate abnormal metaphases. Scale bar, 10 μm. (**g**) Graphs showing percentage of tubules presenting abnormal metaphases at 10- and 16-d.p.p. and in adult *Meioc*^*+/+*^ (white columns) and *Meioc*^*−/−*^ (black columns) testes (left) and percentage of abnormal metaphases cells in 15.5-d.p.c. ovaries (right). Mean±s.e.m., mice analysed *n*=3–6, **P<0.01; ***P<0.001 (Student's *t*-test).

A striking phenotype was observed in both male and female *Meioc*^*−/−*^ meiocytes with the occurrence of numerous abnormal metaphase cells (Fig. 7 and Supplementary Fig. 7). In wild-type gonads such cells are rare and can only be observed at low level and transiently when the first meiotic cells appear (that is, at 14.5 d.p.c. in the ovary and 10 d.p.p. in the testis). These abnormal metaphases were characterized by 40 rounded masses of DNA that were not apoptotic bodies, as indicated with TUNEL staining (Supplementary Fig. 7b). These cells showed histone H3 phosphorylation^36^, a microtubule spindle and the presence of meiotic markers such as SYCP3 and γ-H2AX (Fig. 7a–d and Supplementary Fig. 7d). These last markers indicated that these cells likely engaged meiosis, although they are distinct from normal meiotic metaphases that are negative for γ-H2AX (Fig. 7b). Such abnormal metaphases were retrieved as early as 15.5 d.p.c. in the *Meioc*^*−/−*^ fetal ovary and starting at 10 d.p.p. in the *Meioc*^*−/−*^postnatal testis. In testes, these were frequently observed in tubules containing mostly preleptotene or leptotene cells (Fig. 7e,g). BrdU incorporation assays were performed in *Meioc*^*−/−*^ testes during 36 h to label replicative DNA. The presence of BrdU in the abnormal metaphases suggests that these cells were at pre-meiotic S phase shortly before reaching a metaphase-like stage (Fig. 7f and Supplementary Fig. 7c). These results suggest that, although *Meioc*-deficient meiocytes could initiate MPI, some rapidly and prematurely exit MPI and enter an abnormal metaphase. The observation of such abnormal metaphases in the double mutant *Spo11*^*−/−*^*;Meioc*^*−/−*^ indicates that these are unrelated to meiotic DSB (Fig. 6d). Intriguingly, such abnormal metaphases were similar to those described in *Stra8*^*−/−*^ mice with mixed genetic backgrounds^10^. Given that *Meioc*^*−/−*^ and *Stra8*^*−/−*^ mice share several common features, we postulated that MEIOC may be involved in the implementation of the meiotic programme similar to STRA8.

### MEIOC prevents meiotic mRNA degradation

**Figure 8:**
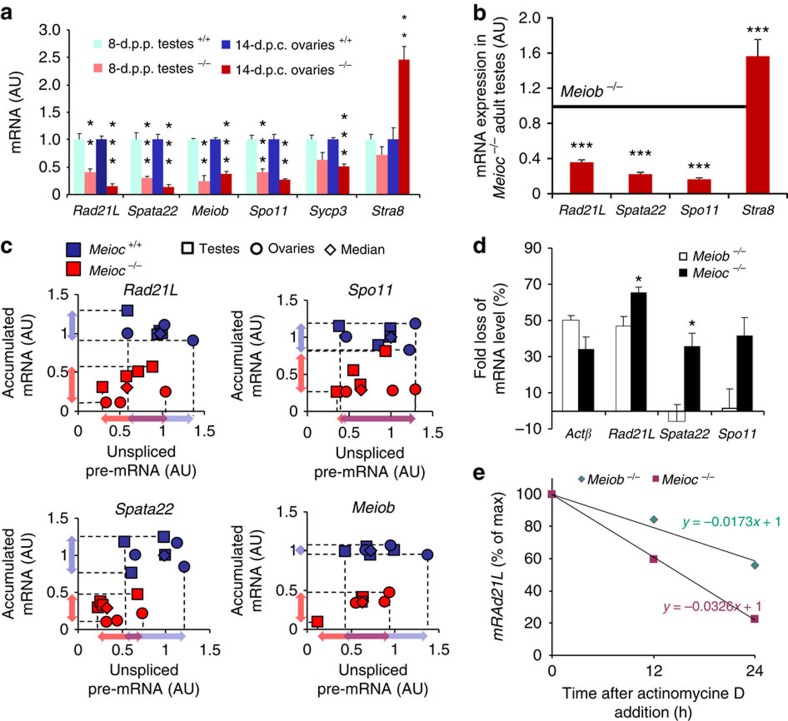
Meiosis prophase I genes are less stable in *Meioc*^*−/−*^ gonads. (**a**,**b**) RT–qPCR measurements of MPI transcripts in whole *Meioc*^*+/+*^ and *Meioc*^*−/−*^(**a**) 14.5-d.p.c. ovaries and 8-d.p.p. testes; (**b**) *Meioc*^*−/−*^ adult testes were compared with *Meiob*^*−/−*^ adult testes as a reference. (**c**) Plot of RT–qPCR data for pre-mRNA and accumulated (mature) mRNA in *Meioc*^*+/+*^ and *Meioc*^*−/−*^ fetal ovaries and postnatal testes. Dotted lines, projections of minimal and maximal values. (**d**) Fold loss of mRNA levels was measured after pulse chase ethynyl uridine incorporation performed in *Meioc*^*−/−*^ and *Meiob*^*−/−*^ adult testes. (**e**) Treatment of testes with actinomycin D to inhibit transcription revealed higher meiotic mRNA degradation levels in *Meioc*^*−/−*^ gonads. The relative expression of *Rad21L* after 12 and 24 h of actinomycin D treatment compared with mRNA expression levels without drug treatment is presented. Linear regressions were calculated, and slope coefficients representing degradation rates were compared between *Meiob* and *Meioc* mutant gonads. Data represent *Rad21L* expression following exposure to actinomycin D. For **a**,**b**,**d**, mean±s.e.m., *n*=3–4; *P<0.05, **P<0.01, ***P<0.001 (Student's *t*-test).

To test whether the apparent deregulation of the meiotic initiation and MPI progression in the absence of MEIOC were caused by alterations in the meiotic transcriptome, we analysed *Meioc*^*−/−*^ ovaries and testes using microarrays. Given that *Meioc* disruption resulted in the elimination of meiocytes during MPI, thereby altering the cellular composition of gonads at an advanced developmental stage, we conducted analyses at early ages when the very first cells initiated meiosis in both sexes, that is, at 14.5 d.p.c. in the embryonic ovary and 8 d.p.p. in the postnatal testis. Analysis of differentially expressed genes revealed that numerous meiotic messengers were downregulated in the mutant gonads (Supplementary Fig. 8). The five most downregulated genes in both sexes were *Rad21L*, *Spata22*, *Terb1*, *Meiob* and *Spo11*, all of which are required for essential processes of MPI^31^,^37^,^38^,^39^,^40^. Altogether, genes affected in both male and female *Meioc*^*−/−*^ mice were mostly downregulated (38 out of 42 affected genes) and half of these genes had already known meiotic functions or were upregulated during meiosis (Fig. 8a,b and Supplementary Fig. 8). This suggests that *Meioc* is mostly involved in the upregulation of meiotic gene expression. These data were confirmed using RT–qPCR at those ages (Fig. 8a). The female transcriptome appeared more severely influenced possibly because of the massive and synchronous meiotic entry occurring at that stage. An even greater loss of most of those meiotic RNA was observed with later testicular stages (Supplementary Fig. 8d) including when *Meioc*^*−/−*^ testes were compared with other mutants with a meiotic arrest such as *Meiob*^*−/−*^ to minimize the difference due to the absence of post-meiotic cells in the adult (Fig. 8b). Among the rare upregulated genes *Stra8* was overexpressed in *Meioc*^*−/−*^ fetal ovaries and in 20-d.p.p. and adult testes, but not in 8-d.p.p. testes (Fig. 8a,b and Supplementary Fig. 8d). This likely represents a change in cell population and this is in agreement with the observed persistence of the STRA8 protein as described above (Figs 4 and 5). Altogether, these results indicate that MEIOC is required for allowing the proper expression of the whole meiotic programme.

To understand the origin of this downregulation, the transcription and stability of meiotic mRNA were investigated in *Meioc*^*−/−*^ testes and ovaries. No significant changes in unspliced pre-mRNA expression were observed between *Meioc*^+/+^ and *Meioc*^*−/−*^ gonads, indicating that transcription was not or was very mildly affected (Fig. 8c). Measurement of RNA degradation with ethynyl uridine indicated that mRNA loss was increased in the absence of *Meioc (*Fig. 8d). Similarly, degradation rates measured after actinomycin D treatment confirmed that meiotic mRNAs were less stable, whereas the stability of *Gapdh* was unchanged in *Meioc* mutants (Fig. 8e and Supplementary Fig. 9a). As aberrant splicing is often the cause for decreased mRNA stability or nonsense mediated RNA decay, we further investigated the existence of alternate transcripts in *Meioc*^*−/−*^ testis for three of the most downregulated genes (*Rad21L*, *Spata22* and *Meiob*) using RT–PCR. Through amplification and sequencing of the full-length mRNAs and of various sites representing potential alternate transcripts (retained intron or nonsense-mediated mRNA decay), we found no change in the splicing of the mRNA (Supplementary Fig. 9b and Supplementary Figs 10–12). These data, thus, suggest that transcription and splicing appeared to take place properly, leading to mature mRNA that were more degraded in *Meioc*^*−/−*^ gonads. Altogether, these data likely explain the pleiotropy of the meiotic defects observed and indicate that MEIOC is involved in the stabilization of meiotic mRNAs, thus highlighting the requirement for an unsuspected mechanism during mammalian meiotic initiation and progression.

### MEIOC interacts with a helicase-binding meiotic mRNAs

**Figure 9:**
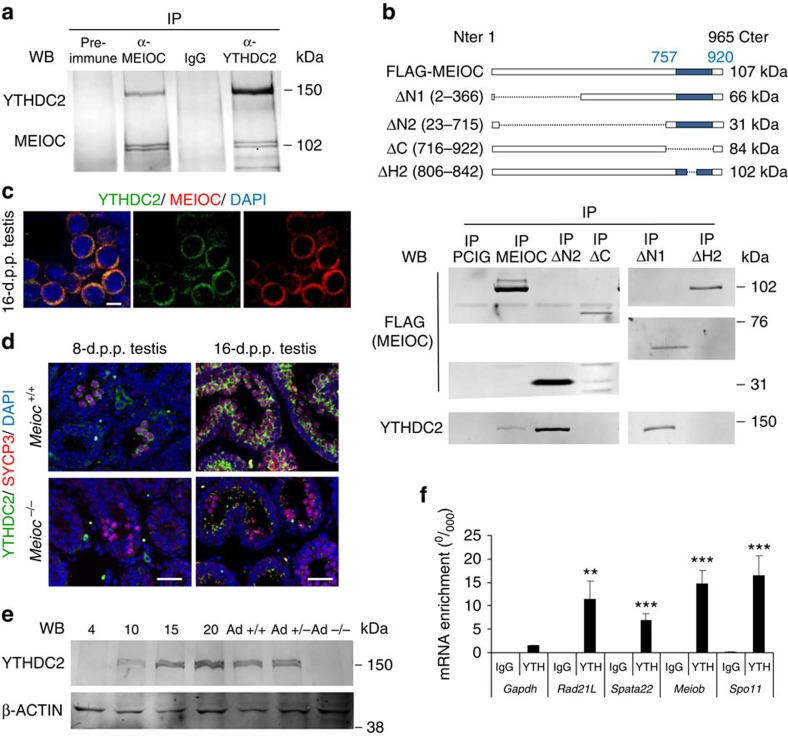
MEIOC/YTHDC2 complex binds meiotic mRNA. (**a**) Western blot analysis of co-IP of MEIOC and YTHDC2 in postnatal testes. (**b**) MEIOC coiled-coil domain interacts with YTHDC2. Upper panel: schematic representation of FLAG-tagged MEIOC complete protein and deletion mutants overexpressed in HEK-293 cells. Numbers in square brackets indicate the deleted amino acids, which are also represented with dotted lines. Blue boxes indicate the coiled-coil domain. Lower panel: co-IP was performed with anti-FLAG antibody, and samples were subjected to western blotting with anti-FLAG and anti-YTHDC2 antibodies. Deletion of the N terminus (ΔN1 and ΔN2) of MEIOC did not abolish the interaction with YTHDC2, whereas deletion of the C terminus of MEIOC (ΔC) or the second helix of the coiled-coil domain (ΔH2) abolished this interaction (**c**). Confocal acquisitions of MEIOC (red), YTHDC2 (green) and DAPI (blue) staining in spermatocytes. Scale bar, 10 μm. (**d**) *Meioc*^*+/+*^ and Meioc^*−/−*^ 8- and 16-d.p.p. testis sections were stained for YTHDC2 (green), SYCP3 (red) and DAPI (blue). Scale bar, 40 μm. (**e**) Western blot analysis of YTHDC2 in mouse testis protein extracts at the indicated postnatal ages (days post-partum). β-actin/ACTB was used as a control. Ad, adult. (**f**) RT–qPCR analysis of mRNA bound after IP assays using anti-YTHDC2 antibody (YTH) or IgG in testicular protein extracts. Statistical analysis compares mRNA fold enrichment/input levels with that of *Gapdh*. Mean±s.e.m.; *n*=6. **P<0.01, ***P<0.001 (Student's *t*-test).

To gain insight into the molecular function of the MEIOC protein, we immunoprecipitated MEIOC-containing protein complexes and analysed their components in young testis (16 d.p.p.) by using mass spectrometry (Supplementary Table 2). The most prevalent protein identified was YTHDC2, an RNA helicase with a YTH domain^41^. This interaction was confirmed in testis extracts using western blot detection with co-immunoprecipitation (IP) of YTHDC2 or MEIOC (Fig. 9a). Notably, this interaction was also observed after benzonase treatment, indicating that the interaction did not depend on the presence of RNA. Using overexpression of various MEIOC mutants in human epithelial kidney cells (HEK-293) cells, we demonstrated that this interaction involved the conserved coiled-coil domain of MEIOC (Fig. 9b). MEIOC and YTHDC2 were co-localized in the cytoplasm of MPI spermatocytes (Fig. 9c). Although *Ythdc2* mRNA is ubiquitously expressed in mammalian cells (for example, gene expression omnibus (GEO) Profiles GDS3834:1779), immunofluorescence and western blot assays indicated that the YTHDC2 protein accumulates in germ cells at the time of MPI, displaying similar spatial and temporal expression to MEIOC (Fig. 9d,e). Interestingly, the YTHDC2 protein was hardly detected in postnatal and adult *Meioc*^*−/−*^ testes, while *Ythdc2* mRNA abundance appeared mildly affected in mutant gonads (Fig. 9d,e and Supplementary Fig. 13). On the basis of these observations, we postulated that MEIOC stabilizes YTHDC2 protein and that the MEIOC/YTHDC2 complex may physically interact with specific target meiotic messengers. RNA-IP assays conducted in wild-type testes with an anti-YTHDC2 antibody indicated that *Meiob*, *Spata22, Spo11* and *Rad21L* mRNA were robustly bound to YTHDC2 (Fig. 9f). Similar experiments performed in cell lines (not expressing MEIOC) and in *Meioc*^*−/−*^ gonads indicated that YTHDC2 bound meiotic mRNA even in the absence of MEIOC (Supplementary Fig. 14a–c). Further experiments in HEK-293 cells overexpressing meiotic mRNA and MEIOC demonstrated that YTHDC2-bound meiotic mRNA was more stable when MEIOC was expressed (Supplementary Fig. 14b,c). We thus concluded that YTHDC2 binds to meiotic mRNAs, allowing their MEIOC-dependent stabilization. Interestingly, in fission yeast, a similar helicase with a YTH domain, Mmi1, binds meiotic mRNA whose stabilization, relying on the interaction between Mmi1 and the meiosis master regulator protein, Mei2, is critical for meiotic entry^42^,^43^. Therefore, selective stabilization of meiosis-specific transcripts at the onset of meiosis seems to be a conserved and critical feature of meiosis from yeast to mammals.

### MEIOC upregulation is independent of retinoid signalling

**Figure 10:**
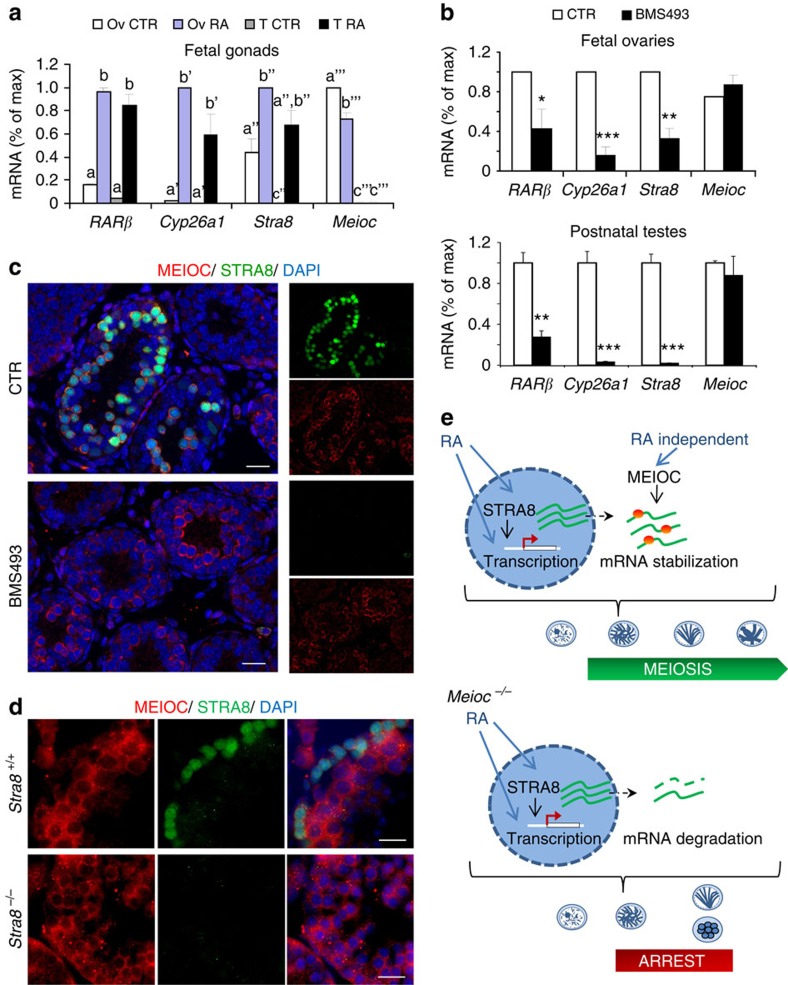
*Meioc* expression is independent of RA and *Stra8* signalling. (**a**) Gonads from 12.5-d.p.c. embryos were cultured and exposed to RA or DMSO. (**b**) Embryonic ovaries (12.5 d.p.c.) and male pups (10 d.p.p.) were exposed to RAR inverse agonist (BMS4693) or DMSO (CTR). For **a**,**b**, RA target genes *Cyp26a1* and *RARβ* (normalized to *β-*actin) and *Stra8* and *Meioc* (normalized to *Vasa/Ddx4* as these are germ cell-specific) expression was measured using RT–qPCR. Mean±s.e.m.; *n*=3–5. For each gene analysed, different letters indicate significantly different data (multiple comparisons ANOVA). *P<0.05, **P<0.01, ***P<0.001 (Student's t-test). (**c**) MEIOC (red), STRA8 (green) and DAPI (blue) staining in 10-d.p.p. postnatal testes treated with BMS493 as in **b** (contralateral gonads) or with DMSO (CTR). Scale bars, 20 μm. (**d**) MEIOC (red), STRA8 (green) and DAPI (blue) staining in *Stra8*^*+/+*^ and *Stra8*^*−/−*^ testis sections. Scale bars, 20 μm. (**e**) Hypothetical model for RA-dependent and -independent regulation of the meiotic programme.

Because MEIOC is required to implement the meiotic programme, we sought to determine whether MEIOC induction depended on the known regulators of meiosis: RA and STRA8 (refs 7, 8). To test whether RA induced MEIOC expression we treated embryonic testes with high doses of exogenous RA. Whereas RA did rapidly induce *Stra8* expression, *Meioc* expression remained low in RA-treated testes indicating that RA was not sufficient to induce *Meioc* expression in embryonic male gonads (Fig. 10a). Germ cells that are induced to enter meiosis by RA treatment in embryonic testis do not progress beyond the leptotene/zygotene stage^44^. Because this arrest resembles the *Meioc*^*−/−*^ phenotype, we postulated that incomplete implementation of the meiotic programme because of the absence of MEIOC is responsible for this partial meiosis. Crucially, the inhibition of RA receptor (RAR) signalling with a reverse agonist in fetal ovaries greatly decreased *Stra8* expression without impairing that of *Meioc* (Fig. 10b). *In vivo* treatments of postnatal testes with the reverse RAR agonist resulted in complete lack of STRA8 mRNA and protein without impairing the induction of MEIOC (Fig. 10b,c). Accordingly, MEIOC was also observed in *Stra8*^*−/−*^ postnatal testes (Fig. 10d). These experiments reveal that the upregulation of MEIOC does not depend on RA signalling. This unexpected discovery indicates that timely and robust installation of the meiotic programme in the germline requires at least two distinct yet complementary triggers: an RA-mediated signalling leading to the transcription of meiosis-specific genes, and a previously overlooked and currently undefined RA-independent mechanism that triggers upregulation of MEIOC, resulting in the stabilization of meiosis-specific transcripts (Fig. 10e).

## Discussion

Meiosis is the heart of sexual reproduction, a universal process in the animal kingdom that is obligatory for fertility. However, the regulation of meiosis is only partially understood in mammals. We report here the first description of a new actor that is critical for meiosis and propose that MEIOC confers stability to numerous meiotic mRNAs allowing proper initiation and progression into MPI. This process likely involves an interaction with an YTH domain RNA helicase that binds meiotic RNA. Importantly, the conservation of MEIOC throughout evolution suggests that this mechanism may be a major and ancestral mode of meiotic control.

In this study, we demonstrated that a previously putative gene was indeed encoding for a protein, MEIOC, which was produced exclusively in the germline at the time of meiotic entry and along MPI. Generation of *Meioc*^*−/−*^ mice allowed the demonstration of the absolute requirement of *Meioc* for meiosis and fertility in both sexes. The pleiotropic meiotic defects, including meiotic initiation, synapsis, extensive bouquet stage and maintenance into prophase, fit well with the fact that many meiotic mRNAs are less abundant in mutant meiocytes. A characteristic feature of *Meioc*^*−/−*^ was the abundant abnormal metaphases that seemed to reflect cells that were directed into meiosis, but because of potentially insufficient meiotic equipment, failed to complete meiosis and switch towards a metaphase fate. Indeed, those cells appear to initiate metaphase abruptly just after entering meiosis without having progressed through all the MPI stages.

To the best of our knowledge, such abnormal metaphases have only been described in the *Stra8*^*−/−*^ mice described in ref. 10. Those mutants present the closest similarities to the phenotypes we observed for our *Meioc*^*−/−*^ mice, both models being in a mixed genetic background. Indeed, both *Meioc* and *Stra8* mutants display complete oocyte loss in postnatal ovary, a mild progression into MPI in postnatal testis and the occurrence of abnormal metaphases^10^. Other studies in a C57Bl6 background have described a complete absence of meiosis entry in both male and female *Stra8*^*−/−*^ mice with a decrease in numerous meiotic mRNA^11^,^15^. We thus propose a putative model in which RA and STRA8 would initiate the transcriptional burst launching the expression of meiotic-specific genes that requires an immediate stabilization through an RA-independent mechanism inducing MEIOC expression. Such a hypothesis would explain why artificial RA induction resulted in only 25% of the fetal XY germ cells initiating meiosis and progressing poorly up to the zygotene stage if they lacked a crucial stabilizer for meiotic messengers^44^. In contrast to STRA8 that is transiently produced around meiotic entry, MEIOC remains present all along MPI, suggesting that its action may not only be required for the initial implementation of the meiotic programme but also to sustain the stability of meiotic mRNA along MPI progression.

Our discovery of MEIOC highlights the intriguing question of why early steps of meiosis require stabilization of meiotic transcripts. In this line, it is worth noting that transcription is vastly abolished when germ cells engage MPI^45^,^46^, possibly because of meiotic genome rearrangement being incompatible with transcriptional activity. Therefore, MPI transcripts are produced at the onset of meiosis and would require specific stabilization to perform their functions throughout the prolonged phases of MPI. Another likely explanation is that the aberrant presence of meiotic-specific proteins during the mitotic cycle may impair the faithful transmission of stable genetic information^47^. Thus, mitotic germ cells would need to actively destabilize spuriously produced meiotic transcripts and, at the time of meiosis initiation, require specific factors such as MEIOC that specifically stabilize meiotic transcripts. In fission yeast, Mei2 has been proposed to trigger meiosis by opposing proteins whose role is to specifically destabilize meiotic transcripts during mitotic growth. Stabilization of meiotic transcripts may therefore represent an ancient conserved mechanism^43^. Interestingly, STRA8 is specific to vertebrates^11^ and does appear to be a relatively recent meiotic trigger, whereas MEIOC homologues can be traced as far back as primitive metazoans (for example, cnidarians). This supports the idea of conserved MEIOC-mediated functions in meiosis initiation throughout metazoans.

The existence of such a process critical for meiosis is a major shift in the understanding of meiotic regulation, but obviously leaves numerous open questions. Among these is the existence of a potential mechanism targeting meiotic mRNA to YTHDC2. Does YTHDC2 bind indifferently to all RNA in meiotic cells or do potential motifs or modification on meiotic mRNA confer specific binding? In yeast, the existence of a determinant-selective removal region in meiotic RNA has been proposed to allow YTH helicase to bind^42^. For other mammalian YTH helicases such as YTHDF2, it is rather a methylation mark that allows specific targeting^48^,^49^,^50^. Consistently, spermatogenic defects were observed in *Alkbh5*^*−/−*^ mice that have an altered mRNA methylation profile^51^. The enzymes and the mechanisms responsible for meiotic mRNA degradation in absence of *Meioc* also remain to be identified. In yeast, this occurs through the exosome but we suspect that XRN1, the major 5′–3′ cytoplasmic exonuclease^52^,^53^, might be involved in mammalian meiotic cells as it is a potential partner for MEIOC based on our co-IP and mass spectrometry experiments. The existence of post-transcriptional control over meiotic RNA stability induced by an RA-independent signal improves our understanding of meiosis regulation, and future investigations are necessary to clarify the exact mechanism at oeuvre.

## Methods

### Mice and embryos

All animal studies were conducted in accordance with the guidelines for the care and use of laboratory animals of the French Ministry of Agriculture and were submitted to the ethics committee of CETEA–CEA DSV (France). Mice were housed in controlled photoperiod conditions (lights on from 08:00 to 20:00) and were supplied with commercial food and tap water *ad libitum*. For dated mating, males were caged with females overnight, and the presence of vaginal plug was examined the following morning. The following midday was defined as 0.5 d.p.c. Mice were killed by cervical dislocation and fetuses were removed from uterine horns before gonad isolation under a binocular microscope. The mice used in this study were NMRI mice (Naval Maritime Research Institute), *Oct4*-GFP, *Spo11*, *Meiob* and *Stra8* mutant mice, which have been previously described^10^,^31^,^40^,^54^, and *Flippase* (129S4/SvJae), *Vasa-cre*^55^ and *Meioc* mutant mice (see below). *Oct4*-GFP mice were used in cell sorting experiments performed with embryonic ovary to determine cell-type specificity^54^,^56^. *Meiob*^*−/−*^ animals were used in some experiments as controls for comparisons with *Meioc*^*−/−*^ because they have similar cell populations^40^. MEIOB is a ssDNA-binding protein mandatory for DSB repair during MPI. *Meiob*^*−/−*^ mice display a meiotic arrest (that is, at pachytene-like stage) and are unable to complete MPI.

### Collection of human fetal gonads

Human fetal material was provided by the Department of Obstetrics and Gynecology at the Antoine Béclère Hospital (Clamart, France) following legally induced abortions in the first trimester of pregnancy and therapeutic termination of pregnancy in the second trimester. Fetal organs were dissected under a binocular microscope, and gonads were removed aseptically and were immediately fixed or lysed^57^. The sex of the fetus was determined by the morphology of the gonads, and the fetal age was evaluated by measuring the length of limbs and feet^58^. Our study was approved by the Biomedicine Agency (reference number PFS12-002), and all women provided informed consent.

### *Meioc* mutant mice generation

The insertion *Meioc* null allele was established at the KOMP Repository (UC Davis, https://www.komp.org) and introduced into JM8N4 C57BL/6N mouse embryonic stem cells. Positive embryonic stem clones were injected into BALB/c N blastocysts, and the derived male chimeras produced germline transmission. Two separate lines were produced. One line was established through crossings with both inbred (C57BL/6) and outbred (NMRI) strains. All lines and strains displayed a similar phenotype. *Meioc*^*+/−*^ mice were crossed with mice carrying ubiquitous *Flippase* (Gt(ROSA)26Sor, Jackson) to remove the LacZ reporter-Neo resistance cassette and generate *Meioc* exon 3 floxed alleles (Supplementary Fig. 4a). *Meioc*^*Flox/Flox*^ males display normal spermatogenesis. Crossings with Vasa-cre mice enabled production of an exon 3-deleted allele (Δ). *Meioc*^*Flox/Δ;*^*Vasa*^*Cre*^ males are phenotypically similar to *Meioc*^*−/−*^ males.

### Real-time quantitative PCR and PCR

Total RNA from pools of whole gonads, purified OCT4-positive or -negative cells or cultured gonads, was extracted using the RNeasy minikit (QIAGEN, Valencia, CA). Reverse transcription was achieved using the high-capacity kit (Applied Biosystems, Foster City, CA) according to the manufacturer's instructions. To measure unspliced RNA levels, a supplemental step with a gDNA eliminator was performed using the RNeasy Plus Mini kit (QIAGEN), and samples without reverse transcriptase (RT) were processed to assess possible genomic contamination. The 7900HT Fast real-time PCR system (Applied Biosystems) and SYBR-green labelling were used for RT–qPCR. The comparative ΔΔcycle threshold method was used to determine the relative quantities of mRNA. β-actin mRNA was used as the endogenous reporter, unless otherwise stated. Each RNA sample was analysed in duplicate. All primers were used at a final concentration of 400 nM. Primers used in the study are shown in Supplementary Table 4. Data are expressed as a percentage of the maximum mRNA expression, unless otherwise stated, in which case an external reference (F9 cells) was used to normalize the expression of numerous data points.

For analysis of alternative splicing, *Meioc*^*+/+*^ and *Meioc*^*−/−*^ 20-d.p.p. testis mRNAs were reverse-transcripted and full-length cDNAs were amplified using PCR with DreamTaq PCR Master Mix (2 × ; Life Technologies). Full-length PCR amplicons were purified with the QIAquick PCR Purification Kit or QIAquick Gel Extraction Kit (QIAGEN) and were submitted to SUPREMErunTM sequencing (GATC). Primers used in the study are shown in Supplementary Tables 4 and 5.

### Protein extraction and western blotting

Protein extracts were produced from cell lines or tissues under native conditions. HEK-293 cells were harvested, centrifuged 5 min at 500 g and then lysed in Cell Lysis Buffer (Cell Signaling, Danvers, USA) complemented with 1 mM 2-mercaptoethanol and complete protease inhibitor (Roche, Mannheim, Germany). For mouse testes, the albuginea was removed and testes were lysed in Cell Lysis buffer using ceramic spheres and FastPrep-24 Instrument (MP Biomedicals) with two pulses of 20 s. Extracts were then gently sonicated (two pulses of 30 s), centrifuged 10 min at 14,000*g* and supernatant was used for functional applications.

Before gel migration, protein samples were supplemented with Laemmli buffer and resolved by 8% SDS–polyacrylamide gel electrophoresis (SDS–PAGE). Gels were electrophoretically transferred to polyvinylidene difluoride membranes (Amersham Biosciences, Buckinghamshire, England) before hybridization with appropriate primary antibodies and fluorescent dye (Cy3, Cy5 or equivalent Alexa Fluor 647)-coupled secondary antibodies (Supplementary Table 3). Images were acquired using Typhoon 9400 scanner (Amersham Biosciences). Scans of uncropped images of western blot lanes presented in the main figures are shown in Supplementary Figs 15–17.

### Generation of the anti-MEIOC antibody

In this study, we used two purified (commercial) antibodies and two homemade sera against the MEIOC protein. All produced similar patterns, and sera were the most efficient for immunofluorescence staining. The full-length cDNA encoding MEIOC was amplified from mouse testis cDNA and cloned into the pDEST17 vector (Invitrogen). This vector was overexpressed in *Escherichia coli* to produce the His-MEIOC fusion protein, which was purified under denaturing conditions (8 M urea) on Ni-sepharose beads. After separation on SDS–PAGE, gel slices were mixed with Freund's adjuvants to immunize guinea pigs. Pre-immune sera and antisera (after 100 days procedure) were yielded and used for immunofluorescence and IP (*cf.* respective paragraph in Methods and Supplementary Table 3). All sera and antibodies were validated against MEIOC recombinant protein expressed in HEK-293 cells as positive controls and *Meioc*^*−/−*^ sections as negative controls.

### Histology and immunohistochemistry

Histological sections, germ cell counting and immunohistochemical staining were performed as follows. Gonads were fixed with Bouin's fluid or 4% formaldehyde. The fixed gonads were dehydrated, embedded in paraffin and cut into 5-μm-thick sections. Sections were mounted on glass, dewaxed, rehydrated and stained with haematoxilin and eosin. The gonocytes were identified on the basis of their large, spherical nuclei and clearly visible cytoplasmic membrane. Preleptotene cells were identified based on the appearance of heterochromatin patches in the nucleus. Meiotic cells displayed marked condensation of the chromatin, forming distinct fine threads with a beaded appearance at the leptotene stage and presenting a characteristic crisscross of coiled chromosome threads at the zygotene stage. Germ cells at the pachytene stage showed marked thickening of the chromosome threads and the coiling characteristic of this stage. The chromosome threads appear to be finer and less individualized at diplotene, and the nucleus becomes larger than in the precedent stage. The Histolab analysis software (Microvision Instruments, Evry, France) was used for counting.

Immunohistochemical staining using appropriate antibodies (Supplementary Table 3) were performed as follows. Tissue sections were mounted on glass slides, dewaxed, rehydrated and submitted to antigen retrieval by boiling for 20 min in citrate buffer (pH 6). Endogenous peroxidase activity was blocked by a 10-min incubation with 3% hydrogen peroxide. Slides were then washed in PBS and blocked for 30 min in 2% horse serum. Slides were incubated overnight at 4 °C with primary antibody diluted in PBS/20% blocking buffer. Bound primary antibody was revealed by 30-min incubation with peroxidase-conjugated secondary antibody (ImmPRESS reagent kit, Vector Laboratories) and finally with 3,3′-diaminobenzidine (DAB substrate reagent kit, Vector Laboratories) or VIP (Vector VIP substrate reagent kit, Vector Laboratories). TUNEL staining was performed using an ApopTag Peroxidase In Situ Apoptosis Detection kit (S7100, Millipore) according to the manufacturer's recommendations. For immunofluorescence, 4% formaldehyde-fixed gonads were submitted to antigen retrieval with citrate buffer (pH 6). Sections were then blocked either in 2% horse serum or 10% donkey serum before adding antibodies, and the slides were mounted with the Vectashield 4,6-diamidino-2-phenylindole (DAPI) medium (Vector Laboratories). For immunofluorescence in *Stra8*^*−/−*^ mice, testes were fixed by 4% paraformaldehyde in PBS, kept in the fixative overnight at 4 °C, washed in PBS, dehydrated and frozen in nitrogen vapours, after cryoprotection through immersion in graded (10 and 20%) sucrose solutions. Sections of the treated testes were mounted on glass slides, post-fixed for 10 min in 4% in PBS and blocked in PBS with 0.1% triton and 5% donkey serum before adding antibodies.

Imaging was performed using a Leica DM5500 B epifluorescence microscope (Leica Microsystems) equipped with a CoolSNAP HQ^2^ camera (Photometrics) and Leica MMAF software (Metamorph). Images were processed with the Image J software. Confocal acquisitions were performed using a Leica SPE laser scanning microscope. Images were processed with the Icy software.

### BrdU incorporation and detection in fetal ovaries

For BrdU incorporation, intraperitoneally (i.p.) injections were administered to 14.5-d.p.c. pregnant females or 15-d.p.p. males with 1 mg of BrdU per 20 g mouse body weight. After, respectively, 3 or 36 h, the fetal gonads or postnatal testes were harvested, fixed in formol and processed for histological analysis. BrdU detection in histological sections was performed with the Cell Proliferation kit (RPN20, GE Healthcare).

### *In vivo* and *in vitro* exposure to RA and RAR inverse agonist

Fetal gonads were harvested, and organotypic cultures were performed as previously described^59^. Briefly, fetal gonads were placed on a single Millicell standing insert (HA, pore size: 0.45 μm; diameter: 12 mm). The inserts bearing the explants were floated on 320-μl DMEM/F12 culture medium in 24-well tissue culture plates and incubated at 37 °C in a humidified atmosphere containing 95% air/5% CO_2_. For RA signalling experiments, 12.5-d.p.c. gonads were harvested and separated from the mesonephros. Gonads were cultured in DMEM/F12 medium devoid of retinoids and exposed to exogenous RA and pan-RAR inverse agonist BMS493 (Sigma-Aldrich), diluted to 10 ^2 ^M in dimethylsulphoxide (DMSO) and used at 10^−6 ^M for 6- and 24-h treatments, respectively. Contralateral gonads exposed to the DMSO were used as controls. For *in vivo* treatments of postnatal testes, BMS493 (50 μg g^−1^ per day) or DMSO was administered daily via i.p. injections in males from 7 to 9 d.p.p., and testes were collected at 10 d.p.p. and processed for histology and RT–qPCR analyses. The expression of *Cyp26a1* and *RARβ*, two RA target genes, was measured to control the efficiency of the treatments.

### Immunofluorescence on preparations of chromosome spreads

Spermatocyte chromosome spreads were prepared as previously described with minor modifications^60^. Briefly, diagnostic slide wells were covered with a thin layer of 0.25% H20/NP40, and fresh testis cell suspension was pipetted into the layer before adding three volumes of 1% paraformaldehyde/10 mM Na-Borat pH 9.2 on top. Samples were incubated for 1 h at room temperature in a humid chamber and then dried under a hood. Slides were washed two times for 1 min with 0.4% H_2_O/Photoflo (Sigma-Aldrich) and three times with water. Slides were stained immediately or dried and stored at −20 °C. Spermatocyte preparations were washed with 0.08% PBS-Photoflo 200 (Sigma) and permeabilized with 0.1% PBS-Tween20 before blocking. They were then incubated overnight with the appropriate primary antibodies in blocking solution at room temperature, followed by 1 h of incubation with secondary antibodies at 37 °C (Supplementary Table 3). Slides were mounted with Vectashield DAPI medium. Images were processed and specific structures were quantified with the ImageJ software (Cell Counter plugin).

### Generation of *Meioc* mammalian expression plasmids

DNA corresponding to the complete murine *Meioc* cDNA Flag-tagged in the N-terminal domain of the protein with flanking ClaI/SmaI restriction sites was synthetized (Sigma-Aldrich). ClaI/SmaI digestion enabled insertion into multiple cloning sites of a pCIG plasmid^61^. Deletions were achieved through the CIGEX platform (CEA, Fontenay-aux-Roses). PCR amplifications were performed on a pUC57-Flag-*Meioc* plasmid with specific primers to delete the following regions: [Glu2-Pro366] ΔN1, [Pro23-Ser715] ΔN2, [Tyr716-Lys922] ΔC and [Ile806-Ser842] ΔH2. Mutations were verified by sequencing after amplification and plasmid purification, and the mutated cDNA sequences were subcloned into the PCIG plasmid using ClaI/SmaI restriction sites.

### Cell culture and plasmid transfection

Human epithelial kidney cells (HEK-293, DSMZ, Braunschweig, Germany) were cultivated in high-glucose DMEM (Gibco) containing 15% FBS (Gibco). PCIG, empty plasmid^61^ and plasmids expressing murine *Meioc* (above) and/or human *MEIOB*^40^ were transfected into cells using Lipofectamine 2000 (Invitrogen) according to the manufacturer's instructions.

### Microarrays

For the microarray analysis, 14.5-d.p.c. ovaries and 8-d.p.p. testes from *Meioc*^*+/+*^ and *Meioc*^*−/−*^ mice were dissected and processed for RNA extraction (RNeasy Mini kit, Qiagen) according to the manufacturer's instructions with DNase I step treatment. RNA concentration and integrity were evaluated with the Agilent Bioanalyzer 2100. Hybridization was performed on Affymetrix Mouse Gene 2.0 ST arrays according to the manufacturer's instructions. Two pools were analysed for each genotype and each gender. We sought to constitute pairs of wild-type and KO pools from embryos and pups that were homogenous for developmental stages and from matched littermates. Ten ovaries from five embryos composed each pool of females, and four testes from four pups composed each pool of males. Pairs of fetal ovaries/postnatal testes were dissected from embryos/mice that were previously weighed. For males, testes were weighed, and contralateral testes were fixed in formol for histological analysis. Data from microarray were robust multi-array averaging-normalized. We first performed an unsupervised PCA to control data and applied a classical two-way analysis of variance with interactions to identify differentially expressed genes using Partek GS. Enrichment analysis was performed through the use of IPA. All data have been submitted to NCBI GEO (GSE66880). Lists of genes were analysed and clustered using GO-terms (Gene Ontology; http://amigo.geneontology.org).

### Co-IP assays

For the co-IP assays, testes or HEK-293 cells were lysed under native conditions with homogenization in cell lysis 1 × buffer (Cell Signaling) supplemented with complete protease inhibitor (Roche) and 1 mM beta-mercaptoethanol, followed by two 30-s medium sonication pulses and centrifugation at 16,200*g* for 10 min at 4 °C. For some experiments, the cells were pre-treated for 2 h with 10-μM MG-132 proteasome inhibitor (Sigma-Aldrich; for example, MEIOC mutant co-IP) or protein extracts were submitted to 270 U ml^−1^ benzonase nuclease (Novagen; for example, mass spectrometry). Extracts were diluted four times in binding buffer (25 mM Tris-HCl, pH 7.5, 150 mM NaCl, 10% glycerol, 1 mM EDTA, 1 mM beta-mercaptoethanol, 10 mg ml^−1^ BSA and complete protease inhibitor, Roche). At least 500 μg protein per sample were used for IP using Dynabeads coated with antibody according to the manufacturer's instructions (Life Technologies) or anti-FLAG M2 magnetic beads (Sigma-Aldrich). The protein/bead mixture was incubated for 2 h at 4 °C under gentle agitation before being washed four times with rinsing buffer (binding buffer supplemented with Triton 0.25%). The elution of immunoprecipitated proteins was performed by resuspending the beads in 20 μl of Laemmli buffer and boiling the samples before magnetic separation and western blotting or tandem mass spectrometry analysis. For MEIOC-partner identification, co-IPs were performed with two different antibodies: a commercial antibody targeting MEIOC (Supplementary Table 1) in *Meioc*^*+/+*^ and *Meioc*^*−/−*^ postnatal testes and with anti-FLAG M2 magnetic beads in HEK-293 cells transfected with the FLAG-MEIOC expression plasmid and cells transfected with an empty vector (PCIG). The samples were processed with a nanoLC-LTQ-Orbitrap XL operating system (Thermo) after trypsin proteolysis, as described previously^62^. Enrichment folds were computed based on spectral counts using the PatternLab programme. The mass spectrometry proteomics data have been deposited to the ProteomeXchange Consortium^63^ via the PRIDE partner repository with the data set identifiers PXD003199 (10.6019/PXD003199) and PXD003200 (10.6019/PXD003200).

### RNA-binding protein IP assay

To analyse whether mRNA bound to YTHDC2, testes or HEK-293 cells were lysed under native conditions as described above, with lysis buffer supplemented with RNase Inhibitor (40 U ml^−1^, RNAseOUT, Life Technologies). Then, 100 μl protein extracts were diluted in 800 μl stringent binding buffer (25 mM Tris-HCl, pH 7.5, 200 mM NaCl, 10% glycerol, 1 mM EDTA, 1 mM beta-mercaptoethanol, complete protease inhibitor and 40 U ml^−1^ RNase inhibitor). Antibody was coated on protein G Dynabeads according to the manufacturer's instructions (Life Technologies), and the coated beads were thoroughly rinsed to avoid RNase contamination. Diluted proteins were pre-cleared with beads before the storage of 5% mixture as an RNA level input reference. Pre-cleared proteins were then incubated with the antibody-coated beads at 4 °C for 4 h before being washed six times successively using two buffers (modified binding buffer with 250 mM NaCl, Triton X-100 0.25% and 300 mM NaCl; Triton X-100 0.25% and without protease inhibitors). Then, 10% of the beads were eluted in Laemmli buffer for western blot analysis. The samples were eluted by 30 min of incubation with 100 μl of proteinase K-containing buffer at 55 °C; 1 ml RLT lysis buffer was added to the mixture and the separated magnetic bead mixture was then used for RNA extraction using the RNeasy Micro kit (Qiagen) with a 15-min DNase treatment step. RNA from the 5% input references was also extracted. RNA from the RNA immunoprecipitation procedure was used for RT–qPCR (high-capacity kit).

### RNA decay using 5-ethynyl uridine incorporation

Adult testes were cut into pieces and cultured on inserts (Millipore) at 32 °C in DMEM/HamF12 (Gibco) containing 10% FBS (Gibco). A 12-h pulse was performed by adding 0.2 mM ethynyl uridine to the medium. After washing, a 12-h chase was performed. The samples were collected in RLT buffer after both pulse and chase procedures, and RNA was extracted. Then, the samples were processed with the Click-iT Nascent RNA Capture kit according to the manufacturer's instructions (Life Technologies). RT–PCR was performed directly on the beads using a High-Capacity cDNA Reverse Transcription kit according to the manufacturer's instructions (Life Technologies). The data were normalized to the geometric means of the *Gapdh* and *Sdha* reference genes.

### RNA decay using actinomycin D treatment

Adult testes were cultured as described in the previous paragraph. Actinomycin D was added at 10 μg ml^−1^, and samples were collected in RLT buffer (Qiagen) at 12 and 24 h. The samples were then processed for RNA extraction and RT–qPCR analysis.

## Additional information

**Accession codes:** Transcriptomic data are deposited in NCBI GEO (GSE66880). Proteomics data have been deposited to the ProteomeXchange Consortium with the data set identifiers PXD003199 (10.6019/PXD003199) and PXD003200 (10.6019/PXD003200).

**How to cite this article:** Abby, E. *et al.* Implementation of meiosis prophase I programme requires a conserved retinoid-independent stabilizer of meiotic transcripts. *Nat. Commun.* 7:10324 doi: 10.1038/ncomms10324 (2016).

## Supplementary Material

Supplementary Information Supplementary Figures 1-17, Supplementary Tables 1-5 and Supplementary References
