# Supplementary material for: Implementation of meiosis prophase I programme requires a conserved retinoid-independent stabilizer of meiotic transcripts
Source: Nat Commun. 2016 Jan 8;7:10324. doi: 10.1038/ncomms10324 (PMC4729902; doi:10.1038/ncomms10324)
Supplement: Supplementary Information — Supplementary Figures 1-17, Supplementary Tables 1-5 and Supplementary References [file ncomms10324-s1.pdf]

Supplementary Information

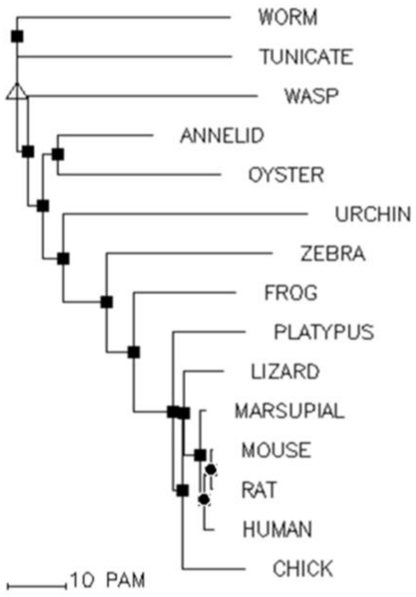

Supplementary Figure 1: MEIOC is a novel evolutionarily conserved protein

Alignments of some representative MEIOC amino acid sequences encoded by orthologs were processed with the Multalin software (http://multalin.toulouse.inra.fr)[1]. Represented species are as follows: worm, *Caenorhabditis elegans*; tunicate, *Ciona intestinalis*; wasp, *Nasonia vitripennis*; annelid, *Capitella teleta*; oyster, *Crassostrea gigas*; urchin, *Strongylocentrotus purpuratus*; zebrafish, *Danio rerio*; frog, *Xenopus tropicalis*; platypus, *Ornithorhynchus anatinus*; lizard, *Anolis carolinensis*; marsupial, *Sarcophilus harrisii*; mouse, *Mus musculus*; rat, *Rattus norvegicus*; human, *Homo sapiens*; and chick, *Gallus gallus*. PAM: percent accepted mutation.

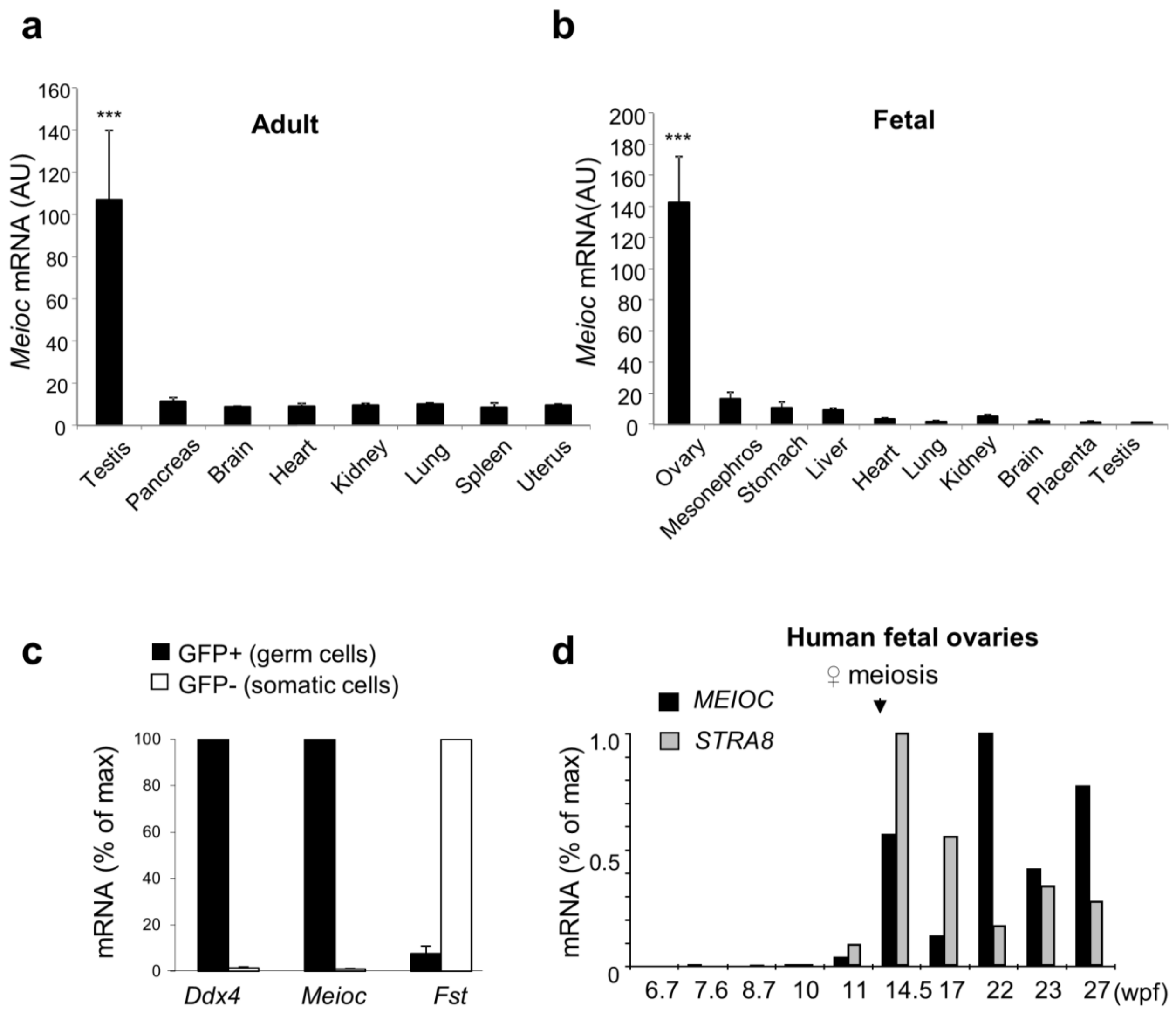

Supplementary Figure 2: *Meioc* is expressed specifically in gonads and meiotic cells

a. b *Meioc* mRNA expression was measured by RT-qPCR in the various indicated adult (**a**) and fetal (**b**) mouse organs. Embryos were collected at 13.5 dpc. Mean±SEM; mice analyzed n=3; *** p<0.001 (Multiple comparisons ANOVA). **c.** *Vasa/Ddx4*, *Fst* and *Meioc* expression was measured by RT-qPCR in the purified germ cell fraction (GFP+) and somatic cell fraction (GFP-) from *Oct4-Gfp* 13.5 dpc ovaries. *Vasa/Ddx4* and *Fst* are specific markers for the germ and somatic populations, respectively. Mean ± SEM, n=4. **d.** *MEIOC* and *STRA8* mRNA expression was measured in whole human fetal ovaries harvested at the indicated developmental stages. wpf, weeks post-fertilization. *MEIOC* expression in human fetal ovaries at the time of MPI reinforces the correlation observed in mouse gonads.

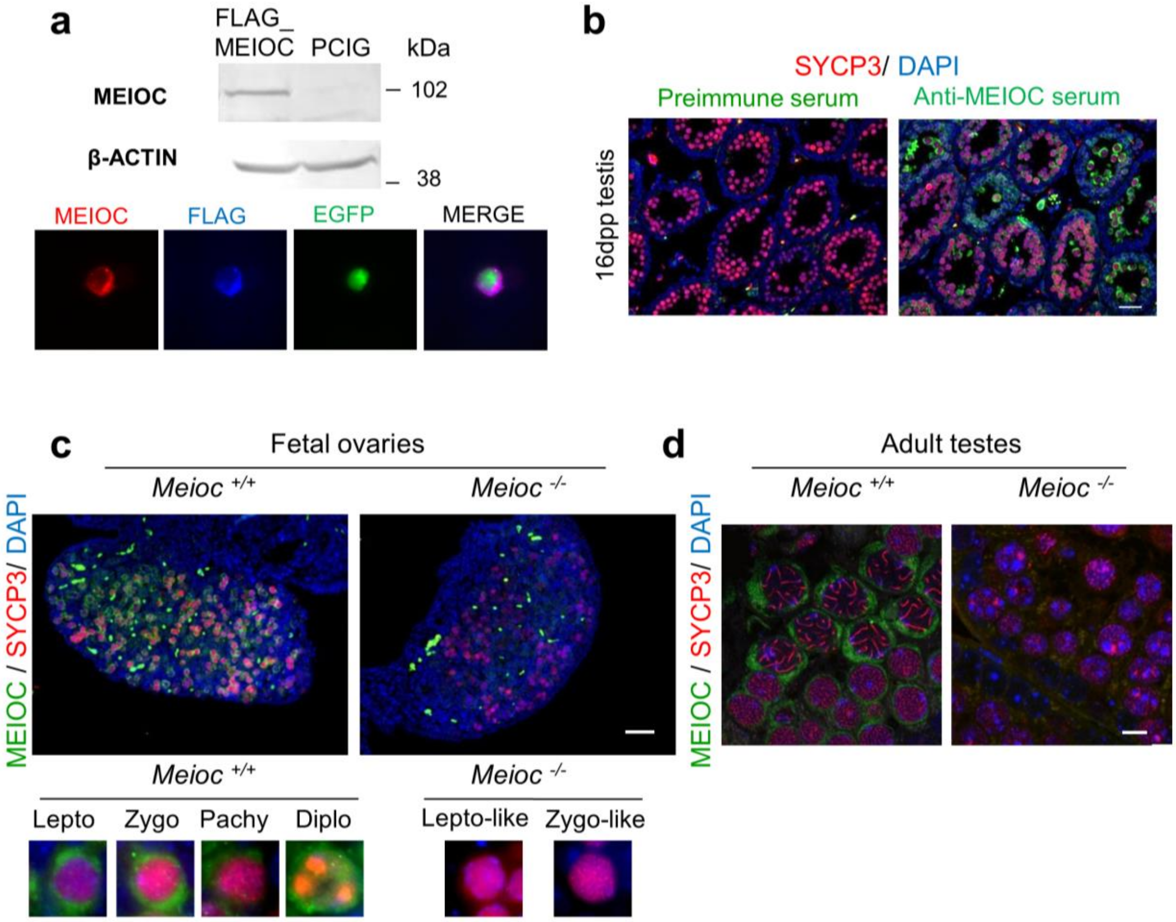

Supplementary Figure 3: Validation of anti-MEIOC sera and antibodies

In this study we used 2 purified (commercial) antibodies and 2 home-made sera against the MEIOC protein. All provided similar results but serum n°2 was the most efficient for immunofluorescence staining. **a.** Anti-MEIOC antibody recognized specifically the recombinant protein. HEK293 transfected with tagged-MEIOC-IRES-eGFP (FLAG_MEIOC) expression vector or empty vector (PCIG). Western blot analysis allowed detection of a band at the expected molecular weight (109 kDa). Immunofluorescence with anti-MEIOC (red) and anti-FLAG (blue) antibodies evidenced a co-localization only in the GFP-positive cells (green). **b**. 16 dpp testis sections were stained with SYCP3 meiotic marker (red) and with anti-MEIOC or preimmune serum (green) and with DAPI (blue). Bar, 40µm. **c,d**. Immunofluorescence for MEIOC (anti-MEIOC serum n°2, green), SYCP3 (red) and DAPI (blue) in (**c**) fetal (15.5 dpc on upper pictures) *Meioc*$^{+/+}$ and $^{-/-}$ ovaries (bar, 40 µm) and (**d**) 16 dpp *Meioc*$^{+/+}$ and *Meioc*$^{-/-}$ testis sections (bar, 10 µm). Magnifications of meiotic germ cells in **c** were captured from 15.5 dpc (Lepto, Zygo, Pachy and Lepto-like) and 18.5 dpc (Diplo and Zygo-like) *Meioc*$^{+/+}$ and $^{-/-}$ ovaries. Lepto, leptotene; Zygo, zygotene; Pachy, pachytene; Diplo, diplotene.

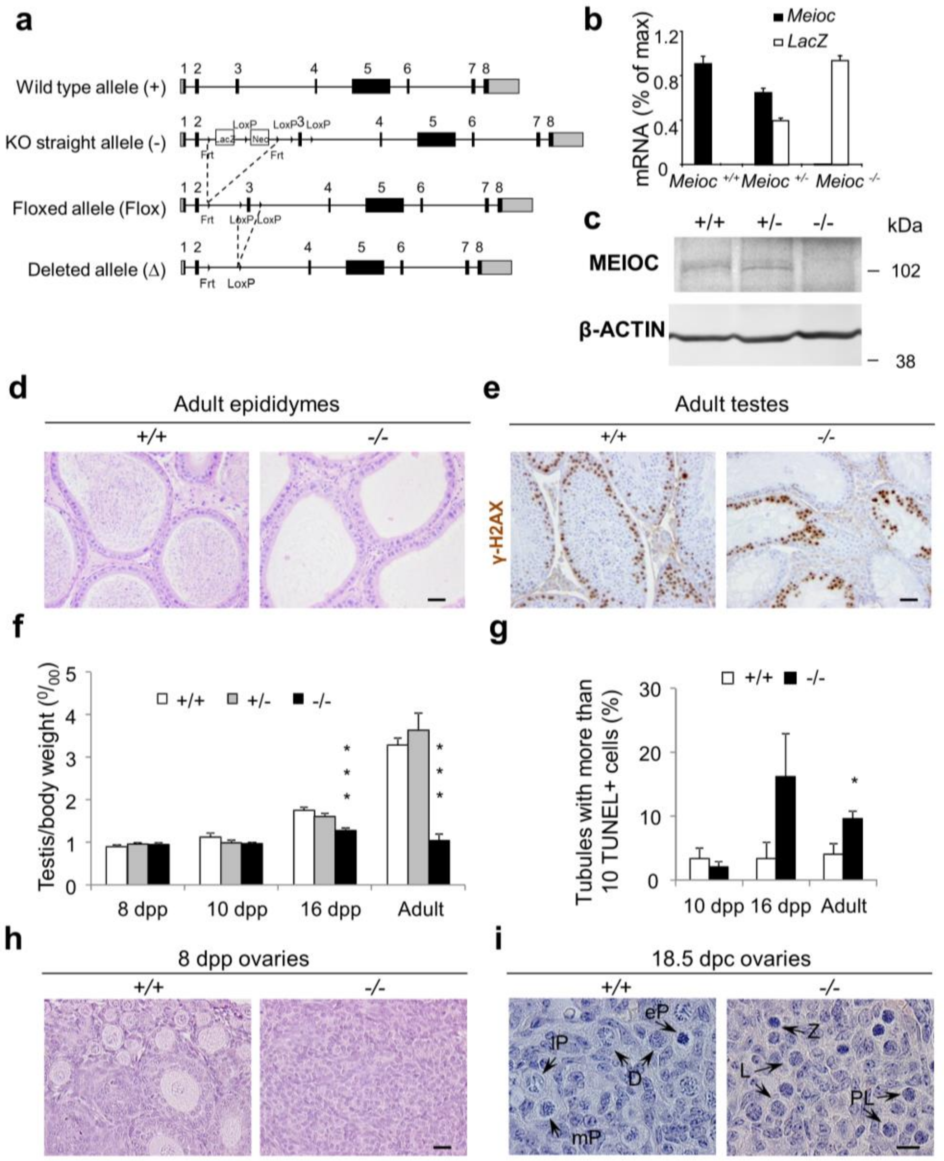

Supplementary Figure 4: *Meioc* invalidation in mice

a. Schematic representation of MEIOC gene invalidation with insertion of a promoter-less lacZ reporter and neomycin selection marker (Neo) between exons 2 and 3 and insertions of two Frt sites and three LoxP sites. This produces a knock-out straight allele due to interruption of the *Meioc* gene (-). Crossings with mice expressing the Flippase and Vasa-cre mice enabled production of a floxed allele (Flox) and an exon 3-deleted allele (Δ). **b**. *Meioc* and LacZ mRNA expression was investigated by RT-qPCR in *Meioc*$^{+/+}$, *Meioc*$^{+/-}$ and *Meioc*$^{-/-}$ adult testes. Mean ± SEM, n=3. **c.** Western blot of *Meioc*$^{+/+}$, *Meioc*$^{+/-}$ and *Meioc*$^{-/-}$ adult testis protein extracts hybridized with anti-MEIOC and β-actin. **d.** Histological sections of adult *Meioc*$^{+/+}$ and *Meioc*$^{–/–}$ epididymides. Bar, 40 µm. **e.** γ-H2AX immunohistochemistry in adult testes exhibiting meiosis prophase I spermatocyte arrest in Meioc-deficient testes. Bar, 40 µm. **f.** The testis/body (mg/g) ratio was calculated for *Meioc*$^{+/+}$ (white columns), *Meioc*$^{+/-}$ (grey columns) and *Meioc*$^{-/-}$ (black columns) mice at 8, 10 and 16 dpp and at adulthood. Mean ± SEM, n=3-26; ***p<0.001 (Student's t-test). **g.** Analysis of the percentage of tubules with more than ten cells positive for the apoptotic marker TUNEL was performed on histological *Meioc*$^{+/+}$ (white columns) and *Meioc*$^{-/-}$ (black columns) at 10 and 16 days post-partum (dpp) and at adulthood. Mean ± SEM, n=3 mice analyzed; *p<0.05 (Student's t-test). **h.** Representative histological sections of 8 dpp *Meioc*$^{+/+}$ and *Meioc*$^{-/-}$ ovaries showing the absence of germ cells in the sections from mutant gonads while oocytes formed follicles in the wild type gonads. Bar, 20 µm. **i.** Representative histological sections showing meiosis prophase I stages in *Meioc*$^{+/+}$ and Meioc $^{-/-}$ 18.5 dpc ovaries. PL, preleptotene; L, leptotene; Z, zygotene; P, pachytene; D, Diplotene; e, early; m, mid; l, late. Bar, 10 µm.

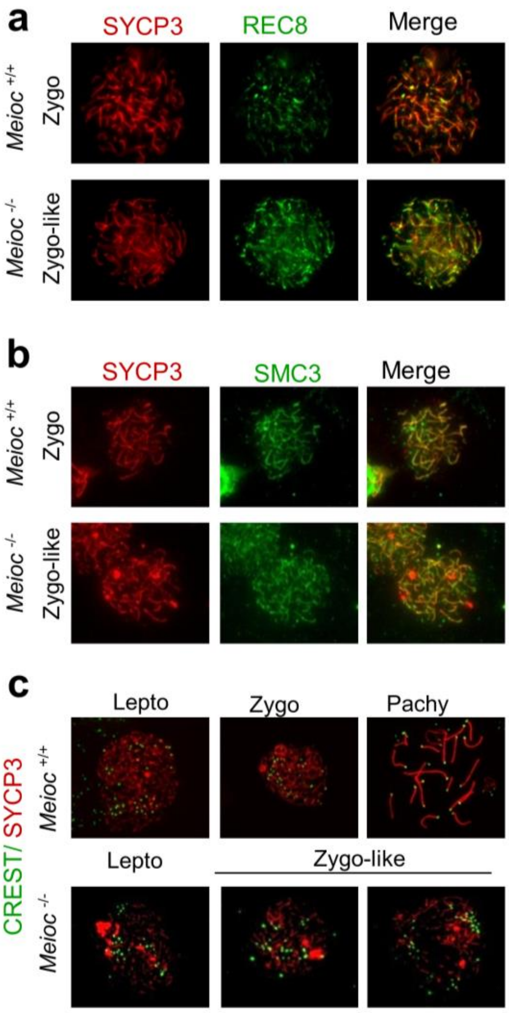

Supplementary Figure 5: Cohesins and CREST localization in *Meioc* deficient spermatocytes

a, b. SYCP3 (red) and (**a**) REC8 (green) or (**b**) SMC3 (green) detection in zygotene *Meioc*$^{+/+}$ and zygotene-like *Meioc*$^{-/-}$ spermatocytes chromosome spreads. Lepto, leptotene; Zygo, zygotene; Pachy, pachytene. **c.** SYCP3 (red) and CREST (green) were detected in the chromosome spreads of various meiosis prophase I wild type and *Meioc*$^{-/-}$ spermatocytes.

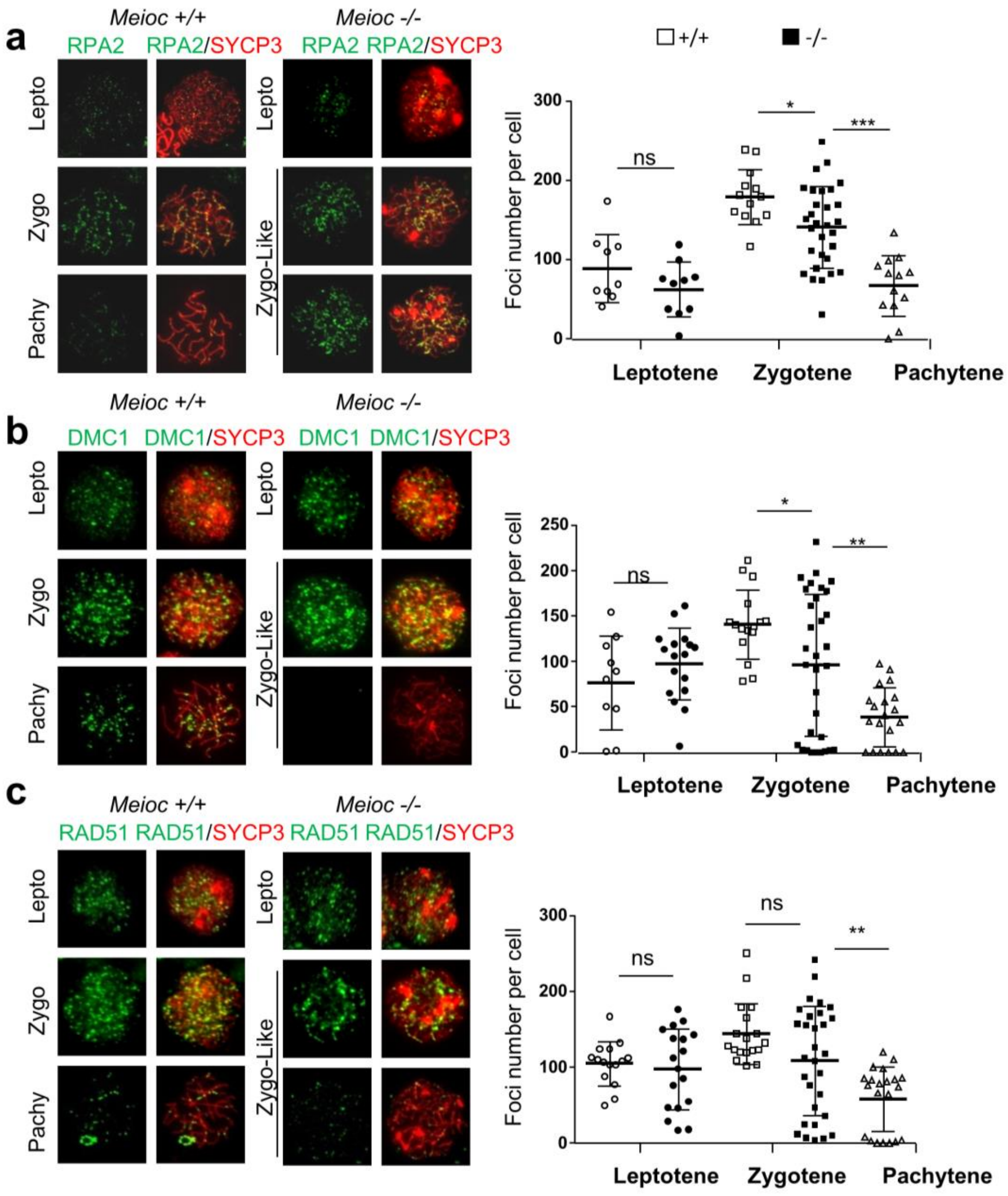

Supplementary Figure 6: Recombination defects in *Meioc* deficient spermatocytes

a-c. Left panels: SYCP3 (red) and RPA2 (**a**), DMC1 (**b**) or RAD51 (**c**) (green, DSB markers) were detected in the chromosome spreads of various meiosis prophase I wild type and *Meioc*$^{-/-}$ spermatocytes. Right panels: Quantification of RPA2, DMC1 and RAD51 foci in wild type spermatocytes at leptotene (n=9; n=10; n=15), zygotene (n=13; n=16; n=18) and pachytene (n=13; n=20; n=21) stages (white symbols) and in mutant spermatocytes at leptotene (n=10; n=17; n=18) and zygotene-like stages (n=31; n=32; n=30) (black symbols). Three mice were analyzed for each genotype. Median numbers of foci are marked by horizontal lines. ns, not significant; *p<0.05, **p<0.01, ***p<0.0001 (Student's t-test).

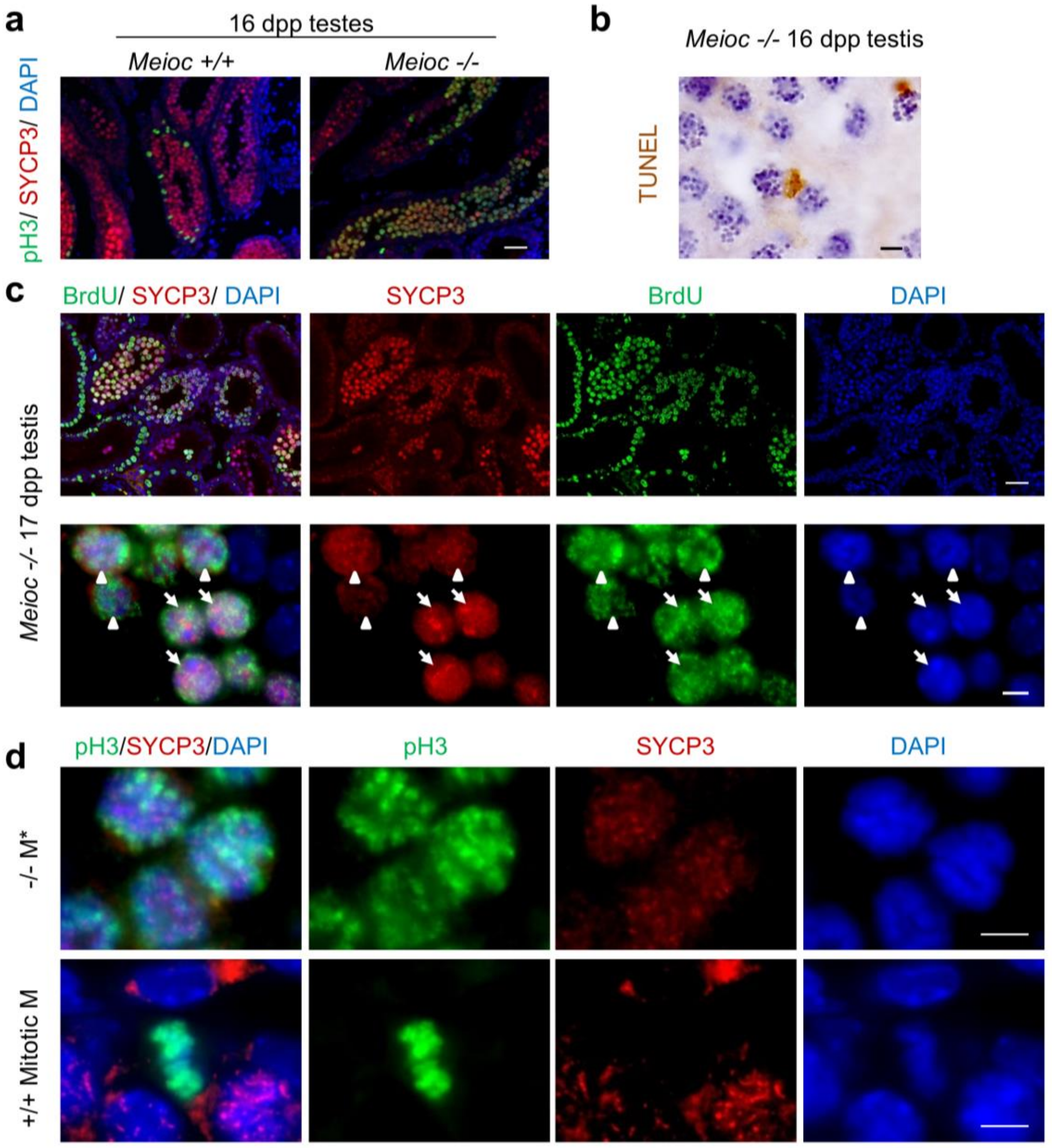

Supplementary Figure 7: Abnormal metaphase phenotype in *Meioc*-deficient mice

a. 16 dpp *Meioc*$^{+/+}$ and *Meioc*$^{-/-}$ testes sections were stained for SYCP3 (red), pH3 (green) and DAPI (blue) revealing abundant abnormal metaphases in *Meioc*$^{-/-}$ seminiferous tubules. Bar, 40 µm. **b**. 16 dpp *Meioc*$^{-/-}$ testes sections were stained for TUNEL. Only few abnormal metaphases are stained with TUNEL apoptotic marker. Bar, 5 µm**. c.** BrdU treated 16 dpp *Meioc*$^{-/-}$ testes sections were stained for SYCP3 (red), BrdU (green) and DAPI (blue). BrdU was injected to 15 dpp *Meioc-/-* mouse 36 hours before harvesting gonads. Upper panel, the presence of BrdU in the abnormal metaphases, preleptotene and leptotene cells suggests that these cells completed premeiotic S phase, bar, 40µm. Lower panel, arrow heads indicate abnormal metaphases and arrows indicate leptotene cells associated in the same tubule. Bar, 5µm. **d.** *Meioc*$^{+/+}$ and *Meioc*$^{-/-}$ testes sections were stained for SYCP3 (red), pH3 (green) and DAPI (blue). Unlike mitotic metaphases, abnormal metaphases in *Meioc* mutants display SYCP3 staining. The abnormal metaphase chromosomes are spherically arranged in rosette configuration instead of being aligned at the cell equator. Bars, 5 µm.

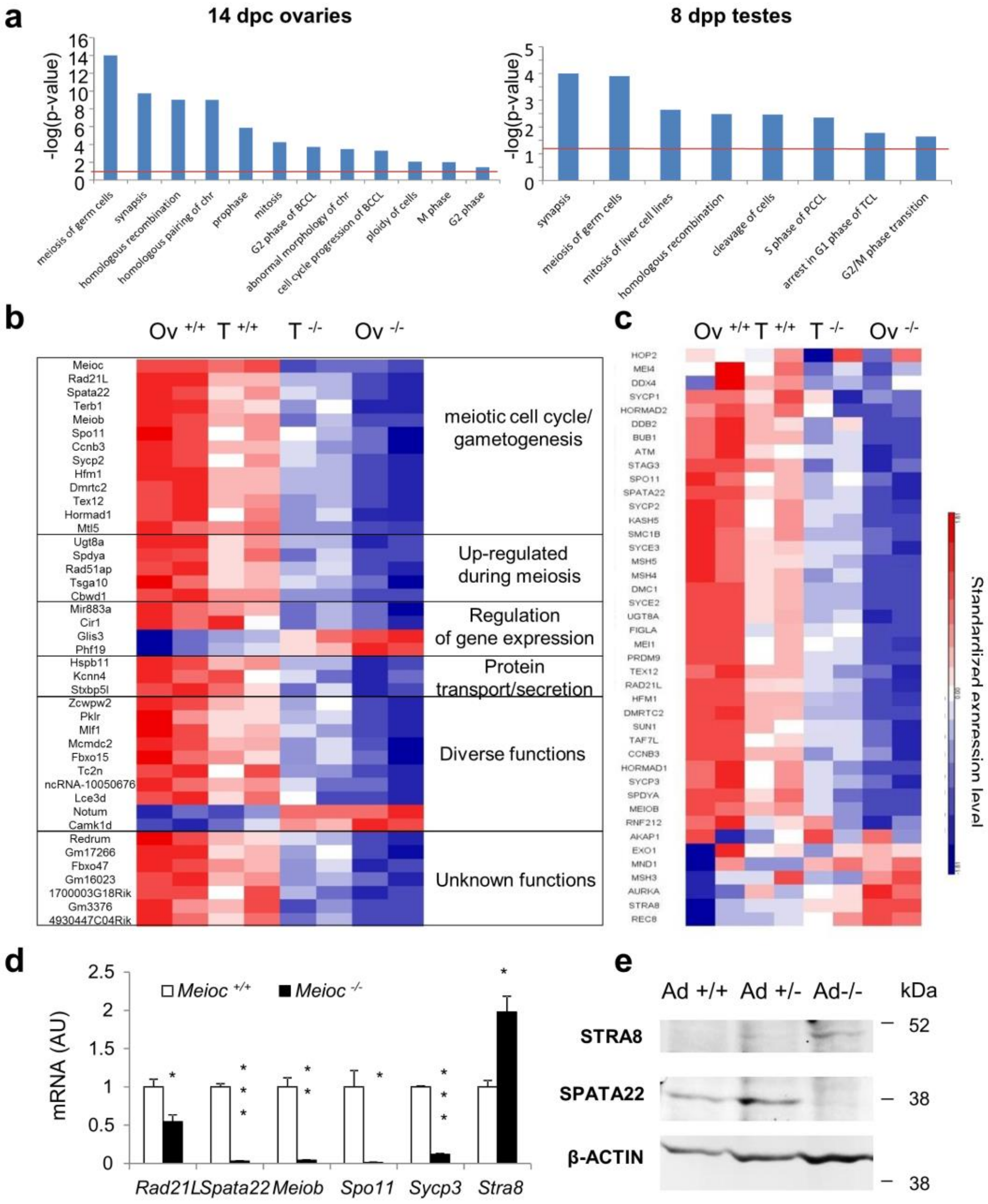

Supplementary Figure 8: Meiosis prophase I genes are down-regulated in *Meioc-/-* gonads

a. Microarray analysis was performed in meiosis initiating male (8 dpp) and female (14.5 dpc) wild type and *Meioc* mutant gonads. Bar charts representing the significantly affected cell cycle functions in meiosis-initiating male and female wild type and *Meioc* mutant gonads. chr, chromosomes; BCCL, bone cancer cell lines; PCCL, pancreatic cancer cell lines; TCL, tumor cell lines. **b.** Heatmap representing the 42 differentially expressed genes in both female and male gonads (Significantly differentially regulated by at least 1.5-fold in mutant fetal ovaries and 1.2-fold in mutant testes ($p<0.05$, ANOVA); Pearson's dissimilarity, mean linkage). Genes were sorted into 6 categories according to the functions identified with GO-term analysis (http://amigo.geneontology.org) and expression profiles (GEOProfile). Ov, ovaries; T, testes. In total, 182 genes were up- or down-regulated by at least 1.5-fold in mutant fetal ovaries, and 114 genes were up- or down-regulated by at least 1.2-fold in mutant testes. **c**. Heatmap representing the differential expression of meiotic genes in both female and male gonads (Pearson's dissimilarity, mean linkage). Ov, ovaries; T, testes. Most of those genes are down-regulated in *Meioc*$^{-/-}$ gonads. Note the exception of *Stra8* and *Rec8* pre-meiotic genes that are not down-regulated in *Meioc* mutants. **d**. RT-qPCR measurements of MPI transcripts in whole *Meioc*$^{+/+}$ and *Meioc*$^{-/-}$ 20 days post-partum testes. Mean ± SEM, n=3-4; *$p<0.05$, **$p<0.01$, ***$p<0.001$ (Student's t-test). **e**. Western blot analysis of STRA8 and SPATA22 in adult mouse testis protein extracts. β-actin/ACTB was used as a control.

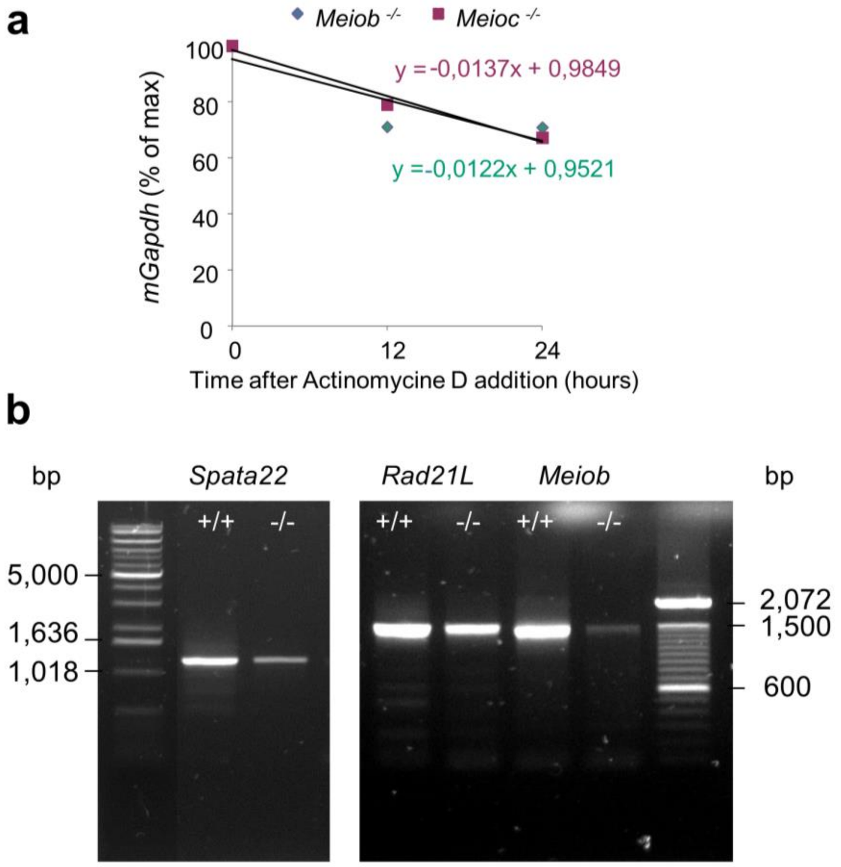

Supplementary Figure 9: **Meiotic transcripts stability and splicing in *Meioc*$^{-/-}$ gonads**

a. *Meiob*$^{-/-}$ and *Meioc*$^{-/-}$ testes were treated with actinomycin D to inhibit transcription. The relative expression of *Gapdh* (as a control for meiotic gene *Rad21L* presented in Figure 8e) after 12 and 24 hours of actinomycin D treatment compared with mRNA expression levels without drug treatment. Linear regressions were calculated, and slope coefficients representing degradation rates were compared between *Meiob* and *Meioc* mutant gonads. Data represent *Gapdh* expression following exposure to actinomycin D. **b.** Alternative splicing was assessed by performing RT-PCR on full length meiotic transcripts in *Meioc*$^{+/+}$ and *Meioc*$^{-/-}$ post-natal testes. Although transcript abundancy was strongly reduced in mutants, no alternate splicing was detected in gel.

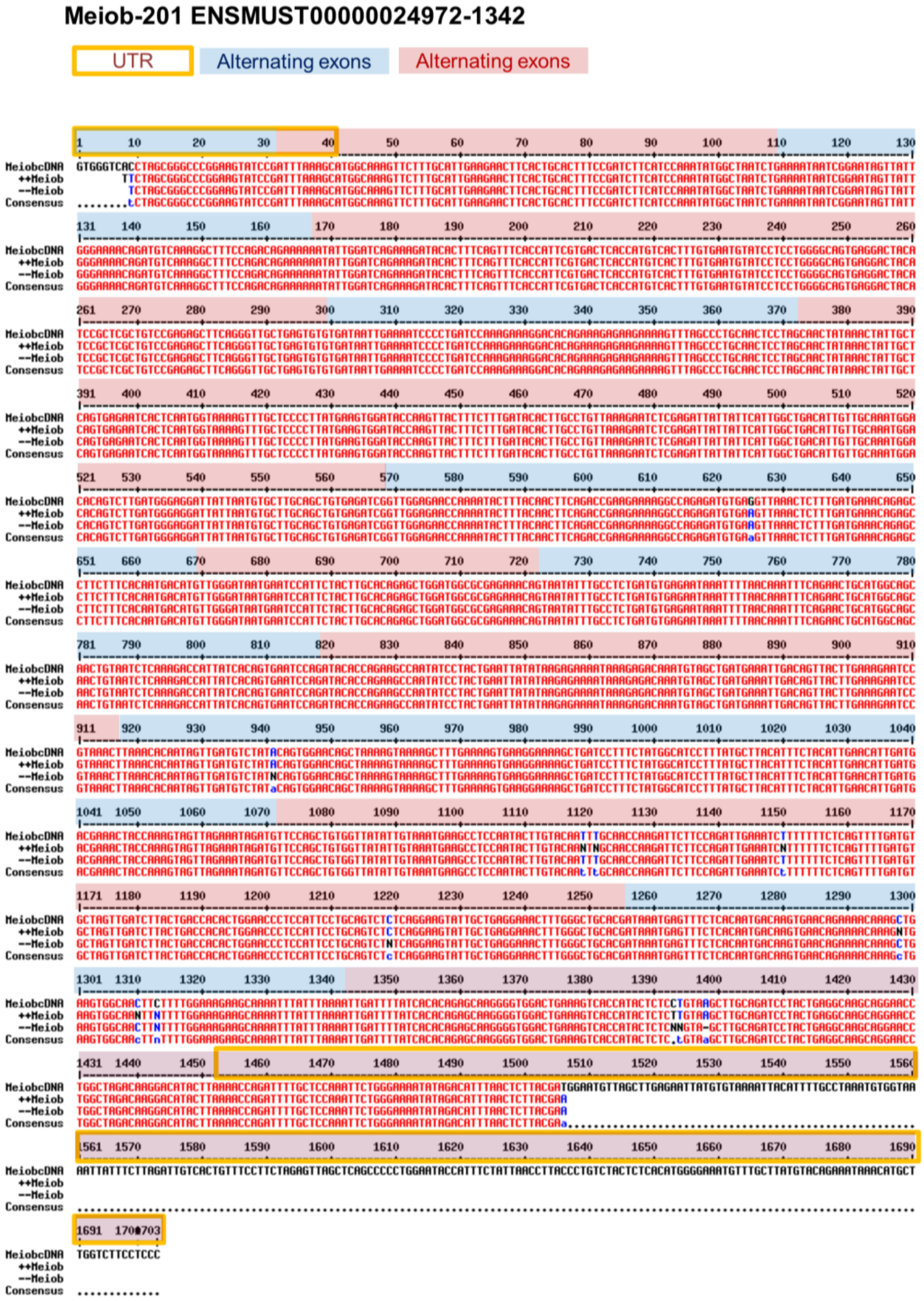

Supplementary Figure 10: ***Meiob* transcript splicing in *Meioc*$^{-/-}$ gonad**

Purified *Meiob* RT-PCR products were sequenced (SUPREMErun^{TM}, GATC, Germany) confirming proper transcript splicing in *Meioc*$^{-/-}$ testes. Sequences from wild type testes (++Meiob) and *Meioc*$^{-/-}$ testes (--Meiob) were aligned with Meiob-201 reference cDNA sequence (MeiobcDNA) from Ensembl Genome Browser (www.ensembl.org). Alignments were performed with Multalin software (http://multalin.toulouse.inra.fr)[1].

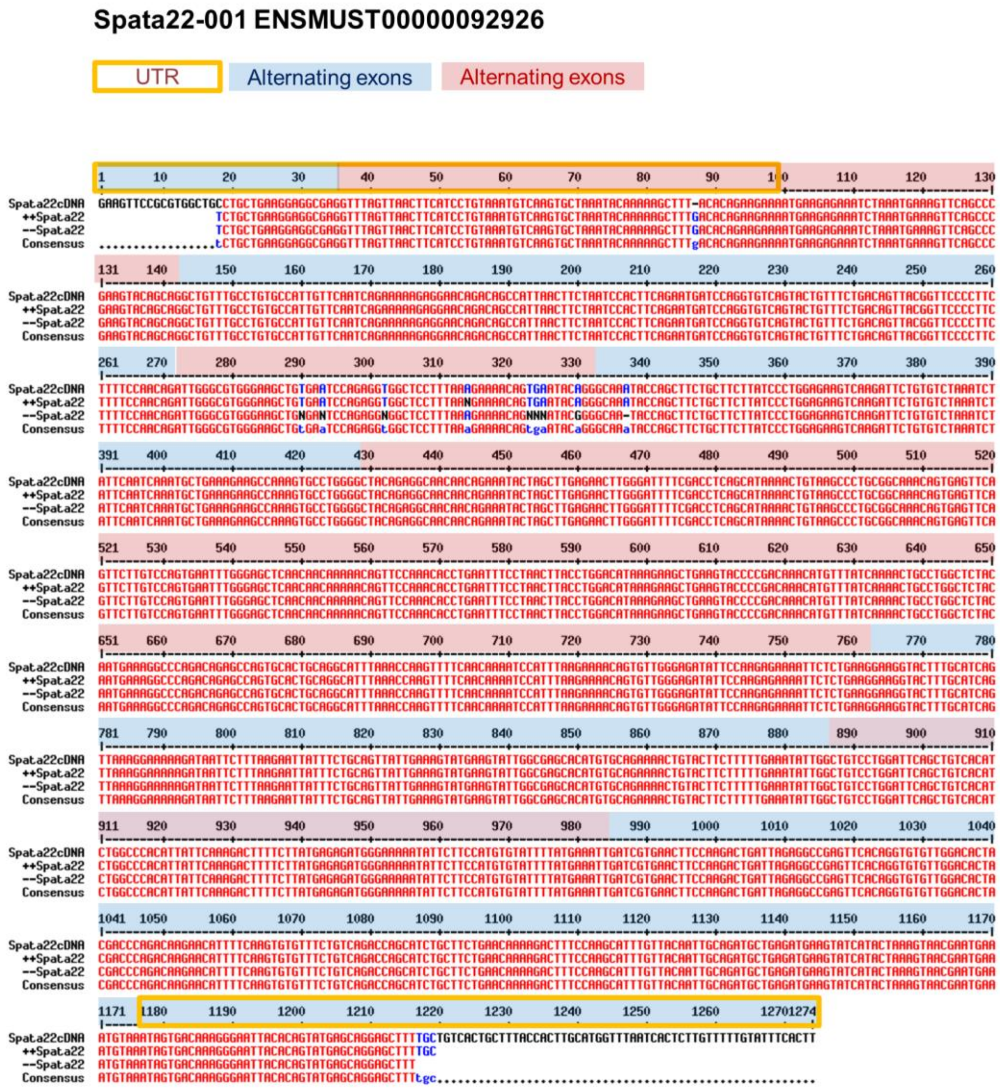

Supplementary Figure 11: ***Spata22* transcript splicing in *Meioc*$^{-/-}$ gonad**

Purified *Spata22* RT-PCR products were sequenced (SUPREMErun^{TM}, GATC, Germany) confirming proper transcript splicing in *Meioc*$^{-/-}$ testes. Sequences from wild type testes (++Spata22) and *Meioc*$^{-/-}$ testes (--Spata22) were aligned with Spata22-001 reference cDNA sequence (Spata22cDNA) from Ensembl Genome Browser (www.ensembl.org). Alignments were performed with Multalin software (http://multalin.toulouse.inra.fr)[1].

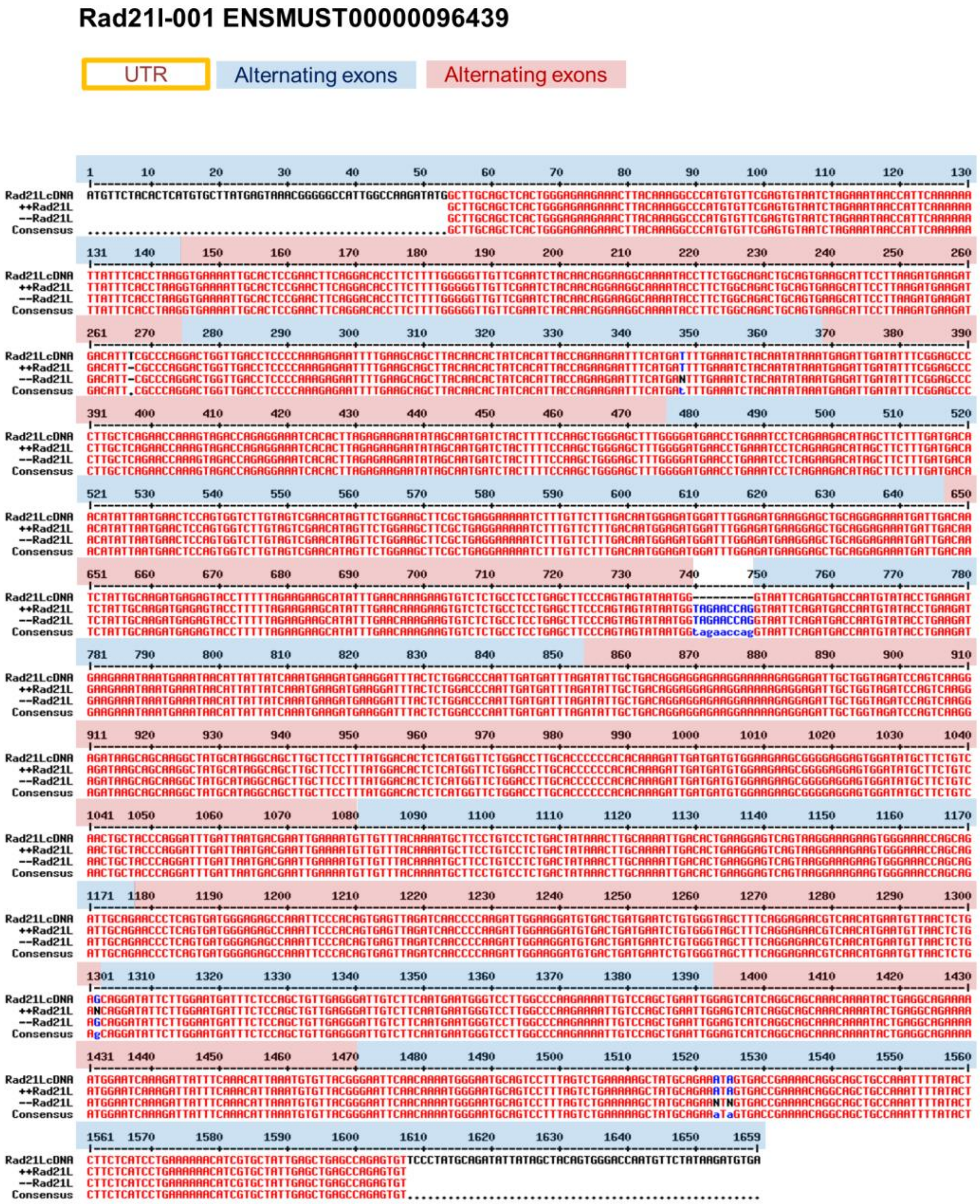

Supplementary Figure 12: ***Rad21L* transcript splicing in *Meioc*$^{-/-}$ gonad**

Purified *Rad21L* RT-PCR products were sequenced (SUPREMErun^{TM}, GATC, Germany) confirming proper transcript splicing in *Meioc*$^{-/-}$ testes. Sequences from wild type testes (++Rad21L) and *Meioc*$^{-/-}$ testes (--Rad21L) were aligned with Rad21L-001 reference cDNA sequence (Rad21LcDNA) from Ensembl Genome Browser (www.ensembl.org). Alignments were performed with Multalin software (http://multalin.toulouse.inra.fr)[1].

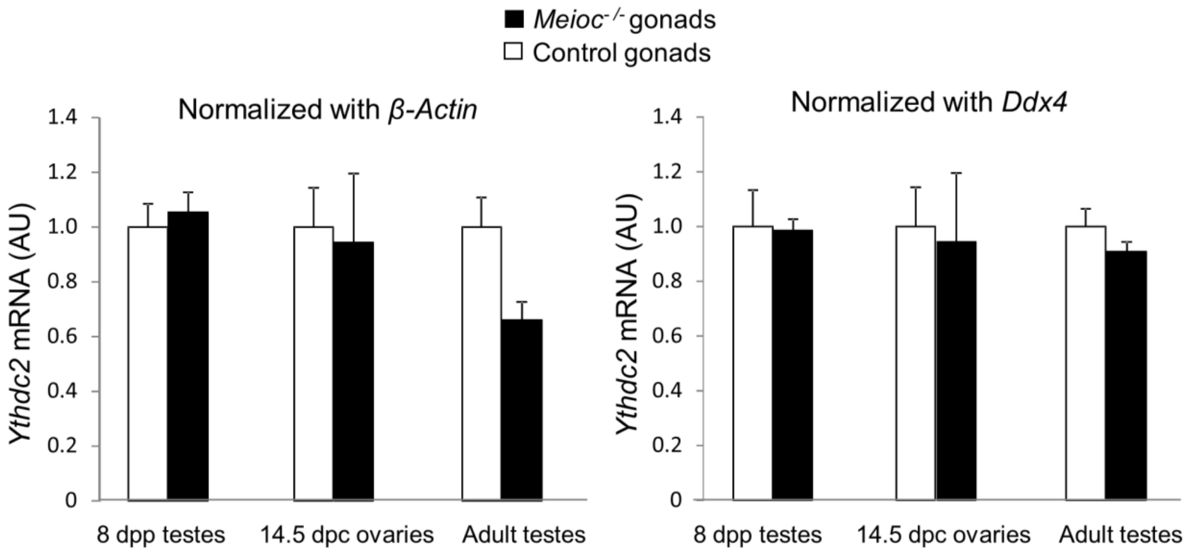

Supplementary Figure 13: *Ythdc2* expression in gonads and *Meioc* mutants

RT-qPCR measurement of *Ythdc2* in whole *Meioc*$^{+/+}$ and *Meioc*$^{-/-}$ 14.5 dpc ovaries and 8 dpp testes and in whole *Meioc*$^{-/-}$ adult testes compared with *Meiob*$^{-/-}$ adult testes as a reference. Right, data were normalized on *β-actin* ubiquitous marker. Left, data were normalized on *Ddx4/Vasa* germ cells specific marker to correct for potential difference in germ cell populations. Mean ± SEM, n=3.

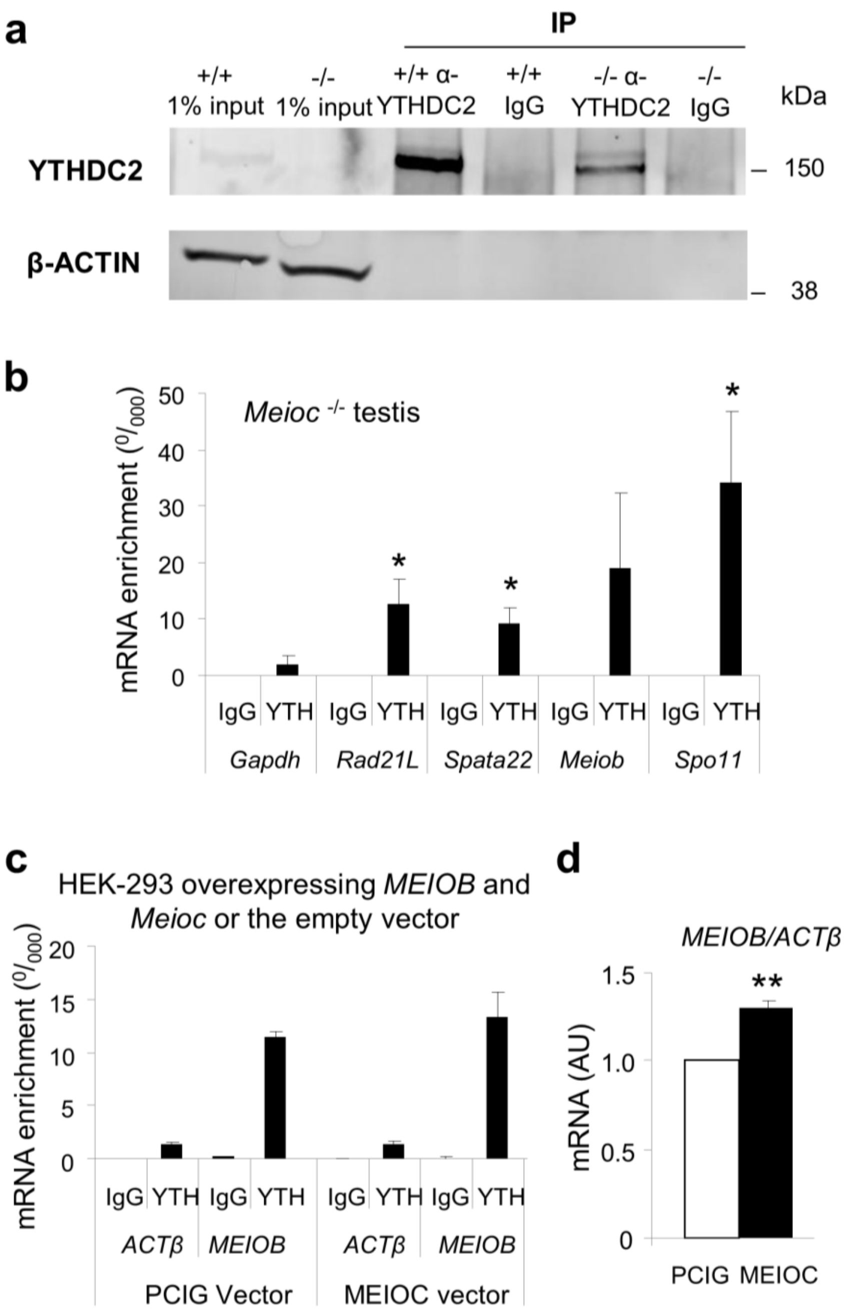

Supplementary Figure 14: YTHDC2 binds meiotic mRNA

a. Western blot analysis of co-IP of YTHDC2 in post-natal *Meioc*$^{+/+}$ and *Meioc*$^{-/-}$ testes reveals that YTHDC2 is immunoprecipitated at a relative low level in absence of MEIOC. **b.** RT-qPCR analysis of mRNA bound after IP assays using anti-YTHDC2 antibody (YTH) or IgG in *Meioc*$^{-/-}$ testicular protein extracts. Statistical analysis compares mRNA fold enrichment/input levels with that of *Gapdh*. Mean ± SEM; n=6. *p<0.05 (Student's t-test). **c.** RIP was performed in HEK-293 cells overexpressing *Meioc* cDNA and *MEIOB* cDNA or PCIG empty vector and *MEIOB,* which was selected as the YTHDC2 target. The *MEIOB* expression plasmid and a *Meioc* containing vector or the empty vector were transfected. Bound *MEIOB* and *ACTB* mRNA were analyzed using RT-qPCR and fold enrichments of mRNA in the RIP samples relative to inputs were calculated. n=3. **d.** MEIOC increases *MEIOB* mRNA levels when over-expressed in HEK-293 cells. *MEIOB* expression plasmid was co-transfected in HEK293 cells with the empty (PCIG) plasmid or with the *Meioc* plasmid (as in **b**) and *MEIOB* expression was measured by RT-qPCR. n=3. Mean ± SEM; **p<0.001 (Student's t-test).

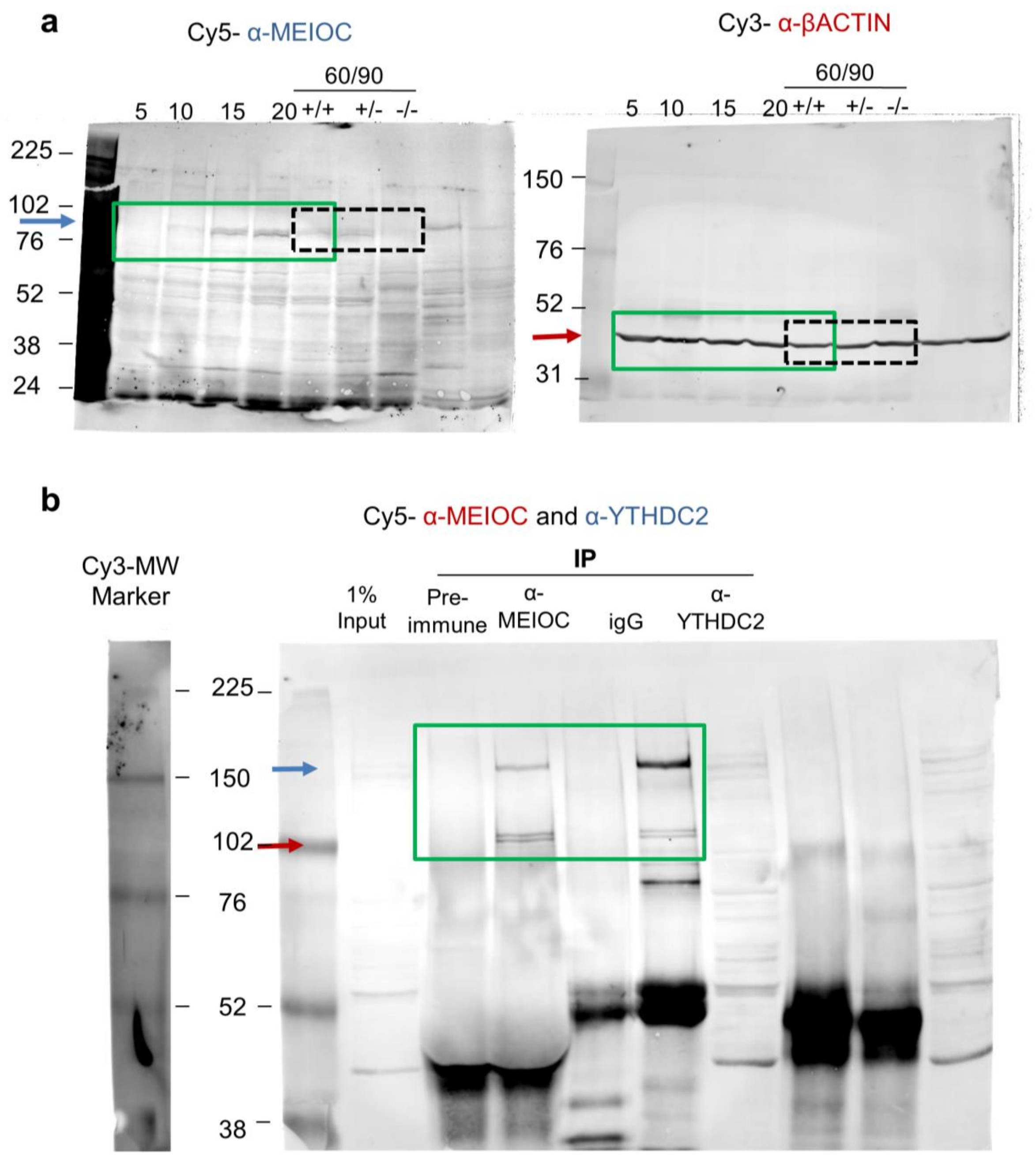

Supplementary Figure 15: Uncropped images of fluorescent immunoblots shown in the indicated Figures

Uncropped images of fluorescent blots displayed in (**a**) Fig 1c and Supplementary Fig 4 ; (**b**) Fig 9a. Green and dotted boxes indicate the lanes shown in the indicated Figures. Cy3 or Cy5 mentions indicate fluorescent dye (or equivalent one) that were used and visualized on the scans. MW: Molecular weight. Numbers on the right of the scans indicate the molecular weight (in kDa) corresponding to the bands of the marker.

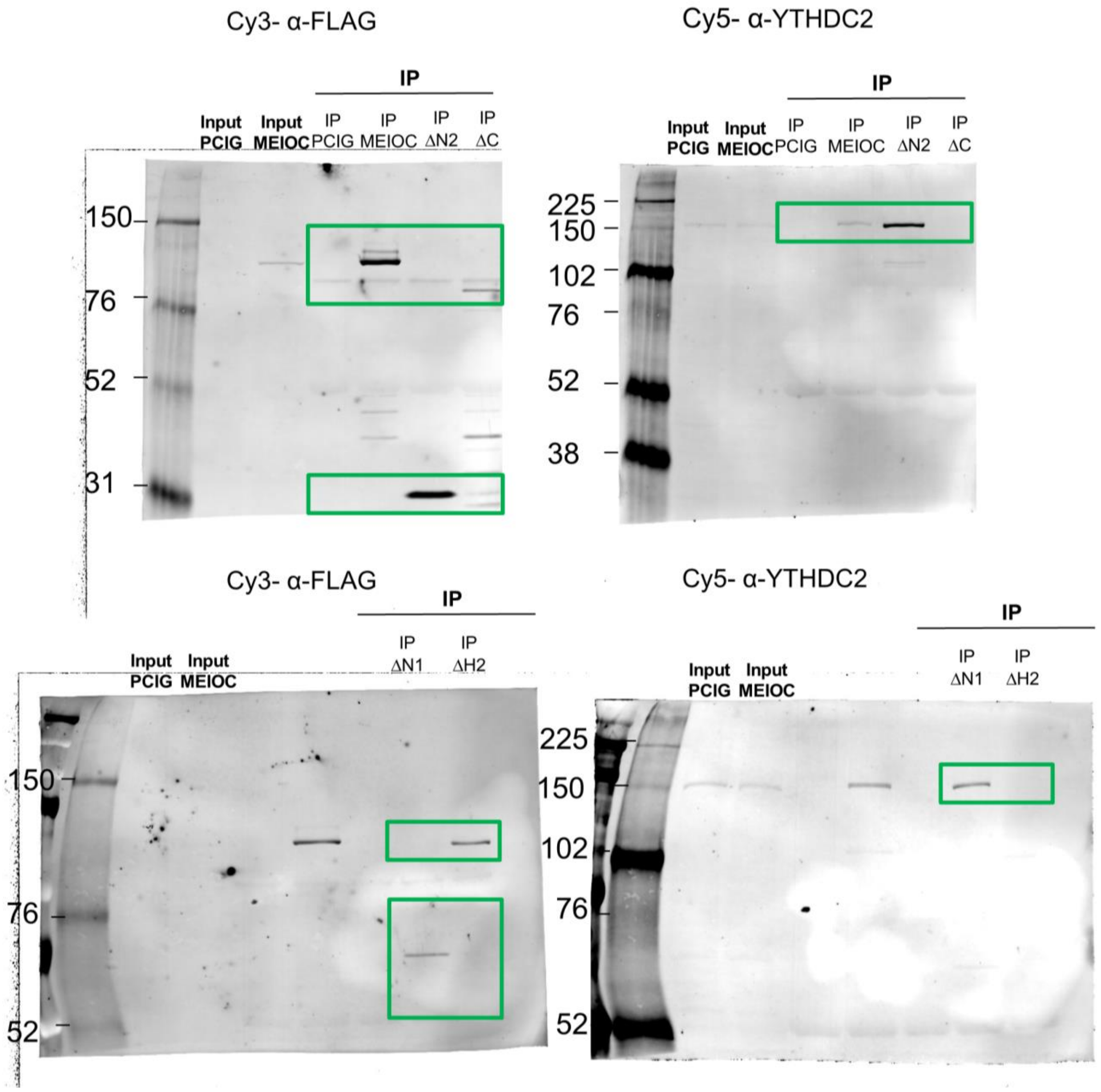

Supplementary Figure 16: Uncropped images of fluorescent immunoblots shown in Fig 9b

Uncropped images of fluorescent blots displayed in Fig 9b. Green boxes indicate the lanes shown in the Figure. Cy3 or Cy5 mentions indicate fluorescent dye (or equivalent one) that were used and visualized on the scans. Numbers on the right of the scans indicate the molecular weight (in kDa) corresponding to the bands of the marker.

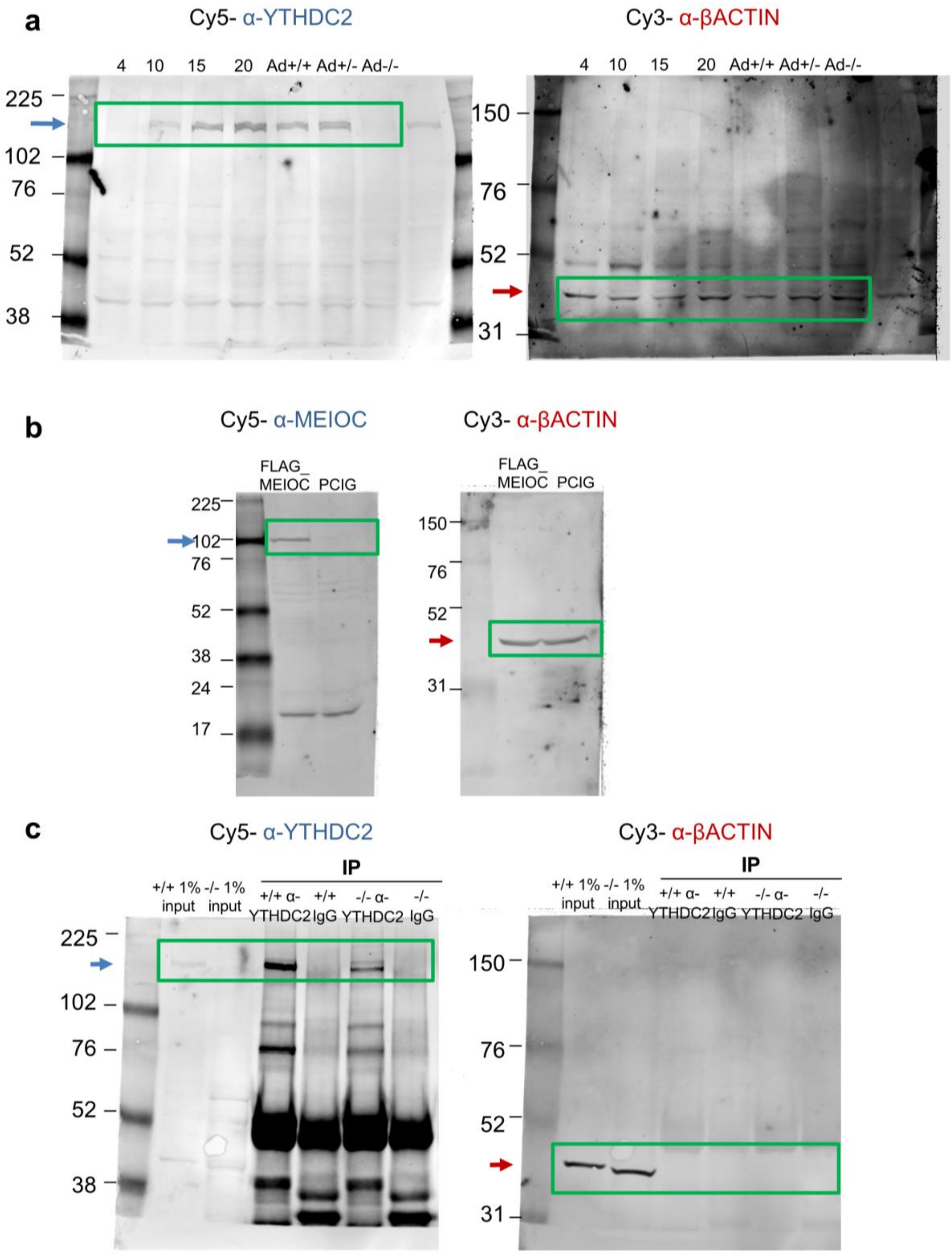

Supplementary Figure 17: Uncropped images of fluorescent immunoblots shown in the indicated Figures

Uncropped images of fluorescent blots displayed in (**a**) Fig 9e ; (**b**) Supplementary Fig. 3a ; (**c**) Supplementary Fig. 10a. Green boxes indicate the lanes shown in the Figures. Cy3 or Cy5 mentions indicate fluorescent dye (or equivalent one) that were used and visualized on the scans. Numbers on the right of the scans indicate the molecular weight (in kDa) corresponding to the bands of the marker.

Supplementary Table 1: MEIOC homologs

Species	Organism	Gene	Protein accession (NCBI / UniProt)
ANNELID	*Capitella telela*		R7UFS9
CHICK	*Gallus gallus*		XP_001234414.2
FROG	*Xenopus (Silurana) tropicalis*		XP_002941590.2
HUMAN	*Homo sapiens*	*C17ORF104*	NP_001138552.2
LIZARD	*Anolis carolinensis*		XP_003222561.1
MARSUPIAL	*Sarcophilus harrisii*		XP_003768468.1
MOUSE	*Mus musculus*	*Gm1564*	NP_001121048.1
OYSTER	*Crassostrea gigas*		K1RFZ3
PLATYPUS	*Ornithorhynchus anatinus*		XP_001517755.2
RAT	*Rattus norvegicus*		NP_001121048.1
TUNICATE	*Ciona intestinalis*		XP_002123724.1
URCHIN	*Strongylocentrotus purpuratus*		XP_003723425.1
WASP	*Nasonia vitripennis*		XP_003426101.1
WORM	*Caenorhabditis elegans*	*Y39A1A.9*	NP_499344.2
ZEBRAFISH	*Danio rerio*		XP_003198174.1

Table specifying protein accessions for MEIOC homologs detected in the represented organisms.

Supplementary Table 2: MEIOC partners identification

Protein	UniProt ID	Accession	Enrichment Fold	Peptides	Function
RNA/nucleotid binding proteins ; RNA processing and translation regulation					
**YTHDC2**	431611	YTDC2_MOUSE	134	38	RNA processing
YBX2	422720	YBOX2_MOUSE	18	6	Translation regulator activity
HSPA2	424783	HSP72_MOUSE	12	14	Protein refolding
PIWIL2	447365	PIWL2_MOUSE	10	6	Translation regulation
HNRNPL	444021	HNRPL_MOUSE	9	4	mRNA processing
EIF4G3	469076	IF4G3_MOUSE	8	4	Translation regulation
LARP7	453908	LARP7_MOUSE	8	5	RNA processing
PRPF8	427142	PRP8_MOUSE	8	7	mRNA splicing
BOLL	469658	BOLL_MOUSE	6	2	Translation regulation
RBMY1A1	468510	RBY1A_MOUSE	6	3	mRNA splicing
SPATA5	435953	SPAT5_MOUSE	6	4	mitochondrion
VIR homolog	476156	VIR_MOUSE	6	4	mRNA splicing
IGF2BP1	477036	IF2B1_MOUSE	5	3	Translation regulation
**XRN1**	459274	XRN1_MOUSE	5	4	RNA processing
RNA/nucleotid binding proteins ; Microtubule organization					
KIF3C	465567	KIF3C_MOUSE	15	2	microtubule-based movement
MARK1	481561	MARK1_MOUSE	12	19	microtubule cytoskeleton organization
TUBA1B	459470	TBA1B_MOUSE	8	8	cytoskeleton
KIF22	478179	KIF22_MOUSE	7	6	microtubule-based movement
SEPT10	435815	SEP10_MOUSE	5	5	cytoskeleton
Ribonucleoproteins; Translation regulation and protein transport					
RPL31	444236	RL31_MOUSE	8	3	ribosome
RPLP1	479103	RLA1_MOUSE	8	2	ribosome
UBAP2L	474843	UBP2L_MOUSE	7	2	ribosome
RPL15	459167	RL15_MOUSE	5	2	ribosome
RPL19	455425	RL19_MOUSE	5	2	ribosome
RRBP1	482113	RRBP1_MOUSE	5	4	endoplasmic reticulum

Proteins in anti-MEIOC IP samples with at least a 5-fold enrichment in wild type 16 dpp testes compared with Meioc$^{-/-}$ samples, as detected by mass spectrometry. Proteins were classified according to their fold enrichment and with GO-terms (http://amigo.geneontology.org). The number of specific peptides identified by spectrometry is provided. Bold indicates proteins also enriched in IP samples performed in HEK-293 cells overexpressing Flag-tagged MEIOC compared with HEK-293 cells overexpressing the corresponding empty vector.

Supplementary Table 3: List of the antibodies used in the study

Antibodies	Company	Ref	Host sp	Mono/Poly	Application	Concentration
MEIOC	Sigma-Aldrich	HPA027266	Rabbit	P	WB ; IF ; IP	1/400 ; 1/200 ; 8µg
β-ACTIN	Sigma-Aldrich	AS41	Mouse	M	WB	1/2500
MEIOC	Toth's Lab		Guinnea-Pig	P	IF ; IP	1/1000 ; 1/10
FLAG	Sigma-Aldrich	F1404	Mouse	M	IF ; WB	1/1000 ; 1/500
STRA8	Abcam	Ab49602	Rabbit	P	IF & IHC ; WB	1/1000 ; 1/400
SYCP3	Abcam	Ab97672	Mouse	M	IF	1/500
P63	Santa-Cruz	Sc-8431	Rabbit	P	IHC	1/200
γH2AX	Millipore	05-636	Mouse	M	IHC ; IF	1/500
DDX4	Abcam	Ab13840	Rabbit	P	IHC	1/200
DDX4	Abcam	Ab27591	Mouse	M	IHC	1/500
POU5F1	Santa-Cruz	Sc-5279	Mouse	M	IHC	1/50
SYCP3	Novus	NB300-232	Rabbit	P	IF	1/400
SYCP1	Abcam	Ab15090	Rabbit	P	IF	1/200
DMC1	Santa-Cruz	Sc-22768	Rabbit	P	IF	1/200
KASH5	Burke's Lab		Rabbit	P	IF	1/500
pH3	Cell Signalling	97065	Mouse	M	IF	1/500
α-TUBULI N	Abcam	Ab80779	Mouse	M	IF	1/500
CREST	Immunovision	HCT-0100	Human	P	IF	1/200
SMC3	Chemicon	Ab3914	Rabbit	P	IF	1/100
REC8	Eijpe et al. 2003 (RαN) [2]		Rabbit	P	IF	1/200
RPA2	Cell signaling	22085	Rat	M	IF	1/200
RAD51	Calbiochem	PC130	Rabbit	P	IF	1/200
YTHDC2	Santa-Cruz	Sc-249370	Goat	P	IF ; WB ; IP	1/200 ; 1/400 ; 8µg
SPATA22	Proteintech Europe	16989-1-AP	Rabbit	P	WB	1/500
Cy5 anti-Rabbit	GE Healthcare	29-0382-78	Goat	P	WB	1/2500
Cy3-anti-Mouse	GE Healthcare	29-0382-75	Goat	P	WB	1/2500
Alexa647anti-Goat	Life technologies	A21447	Donkey	P	WB	1/2500
Alexa488 anti-GuinneaPig	Life technologies	A11073	Goat	P	IF	1/500
Alexa594 anti-mouse	Life technologies	A21203	Donkey	P	IF	1/500
594anti-GuinneaPig	Sigma-Aldrich	SAB4600096	Donkey	P	IF	1/500
Alexa594 anti-rabbit	Life technologies	A21207	Donkey	P	IF	1/500
Alexa488 anti-Rat	Life technologies	A11006	Goat	P	IF	1/500
Alexa488 anti-rabbit	Life technologies	A21206	Donkey	P	IF	1/500
Alexa488 anti-mouse	Life technologies	A21202	Donkey	P	IF	1/500
Alexa350 anti-mouse	Life technologies	A10035	Goat	P	IF	1/500
Alexa488 anti-Goat	Life technologies	A11055	Donkey	P	IF	1/500
Alexa594 anti-Goat	Life technologies	A11058	Doneky	P	IF	1/500
Alexa488 anti-Human	Life technologies	A11013	Goat	P	IF	1/500

Supplementary Table 4: List of all RT-qPCR and PCR primers used in the study

gene	Species	Application	Forward	Reverse
Meioc	Mouse	RT-qPCR	5'-TTAATTGTGGATGAACTTCGAGAACA-3'	5'-GCCCAGTAAAGTCACAACTCTGG
β-Actin	Mouse	RT-qPCR	5'-GCCCTGAGGCTCTTTTCCAG-3'	5'-TGCCACAGGATTCCATACCC-3'
Ddx4	Mouse	RT-qPCR	5'-GAAGAAATCCAGAGGTTGGC-3'	5'-GAAGGATCGTCTGCTGAACA-3'
Fst	Mouse	RT-qPCR	5'-CCAGGCAGGTCCACTTGTGT-3'	5'-AGTCACTCCATCATTTCCACAAAG-3'
Meiob	Mouse	RT-qPCR	5'-ACTACATCCGCTCGCTGTCC-3'	5'-TTATCACACACTCAGCAACCCTG-3'
Gapdh	Mouse	RT-qPCR	5'-CCAGTATGACTCCACTCACG-3'	5'-GACTCCACGACATACTCAGC-3'
Rad21L	Mouse	RT-qPCR	5'-GGTGAAAATTGCACTCCGAAC-3'	5'-CGAACAACCCCCAAAAGAAG-3'
Spata22	Mouse	RT-qPCR	5'-TTCTGACAGTTACGGTTCCCCTT-3'	5'-GCTTCCCACGCCCAATCT-3'
Spo11	Mouse	RT-qPCR	5'-GCCCAGGAGGAGTCTGCAC-3'	5'-CCAGCAATCAATCCCTTGGA-3'
18S	Mouse	RT-qPCR	5'-GAATTCCCAGTAAGTGCGGG-3'	5'-GGGCAGGGACTTAATCAACG-3'
Sycp3	Mouse	RT-qPCR	5'-AAAGAAATGGCTATGTTGCAAAAA-3'	5'-TTGCCACTCCTTGCTGCTGA-3'
LacZ	Mouse	RT-qPCR	5'-ATCCTCTGCATGGTCAGGTC-3'	5'-CGTGGCCTGATTCATTCC-3'
RARβ	Mouse	RT-qPCR	5'-TTTAATCTGTGGAGACCGCCA-3'	5'-TTGTCTACTTTTGTTGGTTCCTCAAG-3'
Stra8	Mouse	RT-qPCR	5'-GCCTGGAGACCTTTGACGA-3'	5'-GGCTTTTGGAAGCAGCCTTT-3'
Sdha	Mouse	RT-qPCR	5'-CCATGACTCTTGAGATCCGTGA-3'	5'-GATCTTTCTCAGGGCCACAGC-3'
Ythdc2	Mouse	RT-qPCR	5'-ATTGATGGCAGGTGATAGCACAT-3'	5'-GAATCGATCCCTTTCATGCACT -3'
*Rad21L **	Mouse	RT-qPCR	5'-TAATCCCCCTGATGATGGCA-3'	5'-TCTCATCATGTTCAAAGGTTCCC-3'
*Meiob **	Mouse	RT-qPCR	5'-ATTTACTGTGAGGGAAAACCCCA-3'	5'-CTCTCCTGAGCAAAGGCTGC-3'
*Spata22 **	Mouse	RT-qPCR	5'-CCCCCCTTTGCCTGTAAGAA-3'	5'-AAATGCTGCTAGGAGTGTGTGTG-3'
*Spo11 **	Mouse	RT-qPCR	5'-GAGACAAACAGAAGCGAGGAGG-3'	5'-GGATGGTGGGATTGCAGATG-3'
MEIOC	Human	RT-qPCR	5'-ATCGGCAAAGGCAAGGAGT-3'	5'-GCGTGTTTTCCGAGTAGCCA-3'
MEIOB	Human	RT-qPCR	5'-CAGAGTCGTCTTTTGCGATAGC-3'	5'-TCGTGGCATCCAGCTCTGT-3'
β-ACTIN	Human	RT-qPCR	5'-GACCCAGATCATGTTTGAGA-3'	5'-TACGGCCAGAGGCGTACAGG-3'
STRA8	Human	RT-qPCR	5'-CTGGACAAAAGTGAGGTTCCG-3'	5'-GGCAAGCACTGAACTGGAGC-3'
Meioc	Mouse	Genotyping	5'-TACCTGCATGCTTTATGTACACCA-3'	5'-TTACAAATAGGCTGCAATTCCTCA-3'
LacZ	Mouse	Genotyping	5'-ATCCTCTGCATGGTCAGGTC-3'	5'-CGTGGCCTGATTCATTCC-3'
Rad21L	Mouse	PCR	5'-ATGGCTTGCAGCTCACTGG-3'	5'-GGAACACTCTGGCTCAGCTCA-3'
Spata22	Mouse	PCR	5'-CTGCTGAAGGAGGCGAGGT-3'	5'-GCAAAAGCTCCTGCTCATACTGT-3'
Meiob	Mouse	PCR	5'-CTAGCGGGCCCGGAAGTAT-3'	5'-TCGTAAGAGTTAAATGTCTATATT-3'

* Pre mRNA primers

Supplementary Table 5: List of all primers used for sequencing of the full length purified PCR amplicons of *Meiob*, *Spata22* and *Rad21L* in *Meioc*$^{+/+}$ and *Meioc*$^{-/-}$ 20 dpp testes

Primers list for sequencing	
cDNA	Meiob
Primer number	3'-5' sequence
P7	AGGATACATTCACAAAGTG
P8	ACAGTTGCTGCCATGCAG
P9	AGCTTACAGGAGAGTATGG
P11	TCGTAAGAGTTAAATGTCTATATT
P21	TGTCCTTTCTTTGGATCAG
P22	TAGCTGTTCCACTGTATAG
P23	TTATCACACAGAGCAAGG
cDNA	Spata22
Primer number	3'-5' sequence
P4	TGCTGAAGGAGGCGAGG
P5	TACTGTGTAATTCCCTTTG
P6	AATCCAGAGGTGGCTCC
P16	ACAGGCAAACAGCCTGC
P17	ATAAGAAGCAGAAGCTGG
P19	TTCAGCTGTCACATCTGG
P27	TACAATTGCAGATGCTGAG
cDNA	Rad21L
Primer number	3'-5' sequence
P2	TCTGGCTCAGCTCAATAG
P3	TACTGACTCCTTCAGTGTC
P13	AATGCAGTCCTTTAGTCTG
P14	TAGTGTTGTAAGCTGCTTC
P24	ATATGCTTCTGTCAACTGC
P26	AAGAATATCCTGCTCAGAG

Supplementary references

1 Corpet, F. Multiple sequence alignment with hierarchical clustering. *Nucleic acids research* **16**, 10881-10890 (1988).

2 Eijpe, M., Offenberg, H., Jessberger, R., Revenkova, E. & Heyting, C. Meiotic cohesin REC8 marks the axial elements of rat synaptonemal complexes before cohesins SMC1beta and SMC3. *The Journal of cell biology* **160**, 657-670 (2003).
